# Engineering a bacterial toxin deaminase from the DYW-family into a novel cytosine base editor for plants and mammalian cells

**DOI:** 10.1186/s13059-025-03478-w

**Published:** 2025-02-03

**Authors:** Dingbo Zhang, Fiona Parth, Laura Matos da Silva, Teng-Cheong Ha, Axel Schambach, Jens Boch

**Affiliations:** 1 Institute of Plant Genetics, Leibniz Universität Hannover, Herrenhäuser Str. 2, Hannover, 30419 Germany; 2 Research Institute of Biology and Agriculture, University of Science and Technology, Beijing, 100083 China; 3 Institute of Experimental Hematology, Hannover Medical School, Hannover, Germany; 4 REBIRTH - Research Center for Translational Regenerative Medicine, Hannover Medical School, Hannover, Germany; 5 Division of Hematology/Oncology, Boston Children’s Hospital, Harvard Medical School, Boston, MA USA

**Keywords:** Crop improvement, Gene therapy, Base editing, CRISPR

## Abstract

Base editors are precise editing tools that employ deaminases to modify target DNA bases. The DYW-family of cytosine deaminases is structurally and phylogenetically distinct and might be harnessed for genome editing tools. We report a novel CRISPR/Cas9-cytosine base editor using SsdA, a DYW-like deaminase and bacterial toxin. A G103S mutation in SsdA enhances C-to-T editing efficiency while reducing its toxicity. Truncations result in an extraordinarily small enzyme. The SsdA-base editor efficiently converts C-to-T in rice and barley protoplasts and induces mutations in rice plants and mammalian cells. The engineered SsdA is a highly efficient genome editing tool.

**Supplementary Information:**

The online version contains supplementary material available at 10.1186/s13059-025-03478-w.

## Background

The discovery of site-specific and programmable DNA binding domains has revolutionized the targeted manipulation of genomes in many different species. First, site-specific nucleases like CRISPR/Cas9, TALEN (transcription activator-like effector nucleases), and ZFN (zinc-finger nucleases) were applied to generate deletions or random mutations at target sites mainly to inactivate specific genes [1–3]. Based on these initial achievements, researchers worldwide endeavored to develop more precise tools that allow for specific nucleotide exchanges to correct human diseases or to generate allelic variants for important crop traits. Targeted base editing is an important development in this direction that resulted in the first CRISPR-based pharmaceuticals approved for clinical treatments in humans to correct single-nucleotide polymorphisms (SNPs) [4–7].

Base editors are typically fusions of Cas9-nickase (nCas9, D10A) as the targeting domain for a specific DNA sequence and an enzymatic domain to deaminate target DNA bases. Based on the hydro-deamination chemistry executed by deaminase enzymes, two main types of base editors: cytosine base editors (CBEs) and adenine base editors (ABEs) have been developed [8, 9]. For CBEs, binding of the CRISPR/Cas complex to a target locus enables the deamination of cytosine to uracil in the displaced single-stranded DNA (the R-loop) [8]. To prevent immediate uracil removement from DNA, an uracil glycosylase inhibitor (UGI) domain is typically fused to the CBE [8, 10]. During replication and repair, the uracil is base-pairing with adenine in the second strand and subsequently replaced by thymine resulting in C-to-T transitions. To trigger transversions (C-to-G) instead of transitions, the base excision repair of uracil can be stimulated by fusing an uracil glycosylase instead of UGI to the nCas9 resulting in abasic sites which can cause random base exchanges [11–13].

In mammalian cells, first APOBEC1 from rat has been used, and more recently, the TadA8e variant of the *E. coli* adenine deaminase TadA has been engineered to alter the substrate preference from deoxyadenosine to deoxycytidine for cytosine base editing [14–16]. In contrast, in plants, APOBEC1 shows particularly low activity [17] and has been replaced by human APOBEC3A (hA3A) [18]. In addition, two other cytosine deaminases, i.e. hAID from human and PmCDA1 from sea lamprey, have been used in plants with variable efficiencies [19–21].

Interestingly, suitable deaminase domains can not only be found in higher eukaryotes but also in bacteria. Recently, a bacterial toxin secreted by *Burkholderia cenocepacia* has been described as a double-strand DNA (dsDNA)-specific cytosine deaminase (DddA). This novel CBE domain allowed the development of TALE-based CBEs [22, 23]. In contrast to the CRISPR/Cas complex, TALEs do not unwind the DNA upon binding to target sites and only produce suitable substrates for dsDNA-specific deaminases as DddA. In addition, TALE-base editors can be imported into cellular organelles using an N-terminal targeting signal, resulting in efficient chloroplast and mitochondrial base editing in plant and mammalian cells, respectively [24–26]. To circumvent DddA toxicity when used in a genome editing tool, DddA has been split into two halves that only complement each other to a functional enzyme upon binding of two matching TALE-base editors with one half of DddA each at a target locus [22, 23, 27]. Non-toxic DddA variants have also been developed to be used with only one TALE [27]. One caveat of DddA is its target requirement for TC motifs [22, 28]. This constraint has been partially relieved through protein evolution into variants with HC (H = A, C, or T) specificity [23]. Applying bioinformatic sequence homology searches, different members of the DddA-family have been identified as CBEs, including ssDNA and dsDNA-specific cytosine deaminases with a broader target specificity [29–31]. Such studies indicate the ongoing need to develop novel base editing domains.

Recently, another bacterial cytosine deaminase, SsdA (single-strand DNA deaminase toxin A), from plant-pathogenic *Pseudomonas syringae* has been described, which is also a type VI-secreted protein that is highly toxic upon expression in bacterial cells (Additional file 1: Fig. S1) [32]. In *P. syringae,* the inhibitor protein, SsdA_I_ binds to SsdA to prevent self-toxicity. SsdA is predominantly active on ssDNA, but has residual dsDNA deaminating activity in vitro and the precise conformations of the deaminase domain SsdA_tox_ in complex with the inhibitor SsdA_I_ has been solved by crystallography [32]. Interestingly, SsdA_tox_ showed a target specificity for Cs with relaxed preference for neighboring pyrimidines, making this a very promising candidate to develop it into a novel genome editing tool.

Here, we have applied SsdA_tox_ as ssDNA-specific deaminase fusion to nCas9-UGI to constitute a novel CBE tool for genome editing. When used for transient expression in *Nicotiana benthamiana*, we experienced toxicity in *A. tumefaciens* even though the used *35S* promoter should only have residual activity in bacteria. Interestingly, we were able to isolate the spontaneous amino exchange mutant SsdA^G103S^, which shows significantly reduced toxicity, but enhanced target deamination. Our study demonstrates that this member of a novel deaminase family can be engineered into a highly efficient CBE tool to generate allelic variants in rice plants. Such enhanced genome editing tools are pivotal for crop improvement to address the challenge of feeding an expanding global population in the looming global warming crisis.

## Results

### Isolation of less toxic SsdA_tox_ variants

**Fig. 1:**
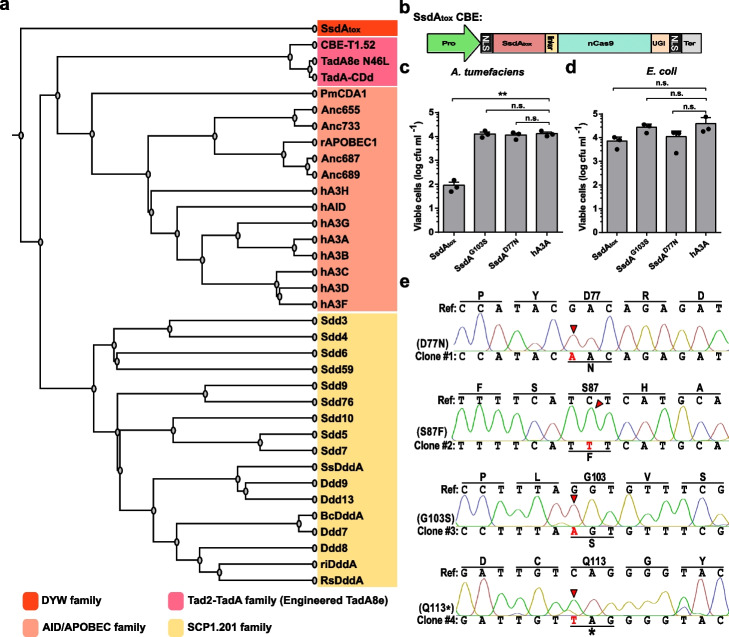
SsdA_tox_-CBEs exhibit mutagenic activity.** a** Phylogenetic tree analysis of ssDNA and dsDNA cytidine deaminases used in cytosine base editors (CBEs). **b** Architecture of the SsdA_tox_ CBE system. Pro: promoter, NLS: nuclear localization sequence, nCas9: SpCas9 D10A nickase, UGI: uracil glycosylase inhibitor, Ter: terminator. **c** Viability in colony-forming units of *A. tumefaciens* GV3101 strains after transformation of binary vectors containing SsdA_tox_-CBE or hA3A-CBE. **d** Viability in colony-forming units of *E. coli* Top10 strains after transformation of binary vectors containing SsdA_tox_-CBE or hA3A-CBE. Values and error bars indicate the mean ± SEM, *n* = 3 independent experiments. ** *P* < 0.01; n.s. (not significant) using Student’s two-tailed unpaired *t*-test. **e** Sanger sequencing of SsdA_tox_ coding region. Mismatches are highlighted in red and are indicated by red triangles. Encoded amino acids are indicated in single letter code above the chromatograms for the reference sequence and below the chromatograms for the mutant variant

SsdA is a bacterial toxin from *P. syringae* with cytosine deaminating activity [32]. Unlike the cytosine deaminases that have been used in CBEs, SsdA is classified into the DYW-family (Fig. 1a) which is a promising class of enzymes to develop genome editing tools. To explore the potential use of SsdA in a CBE, we cloned the catalytic domain of SsdA (SsdA_tox_) from *Pseudomonas syringae* pv. *aptata* and fused it to nCas9-UGI using our genome editing MoClo kit (Fig. 1b; Additional file 1: Fig. S2). We assembled the SsdA_tox_-CBE under control of the 2 × 35S promoter into a level-M binary vector to test its base editing-activity in a GUS reporter system in plants [33]. We noticed that there was a significant (approx. 100x) decrease in colony-forming units (CFU) following transformation of the SsdA_tox_-CBE construct into *A. tumefaciens* in comparison to a hA3A-CBE construct (Fig. 1c). This difference in transformation efficiency was not visible in *E. coli* (Fig. 1d). We speculate that the 2 × 35S promoter has a low background activity in *A. tumefaciens*, but not *E. coli*, producing the SsdA_tox_-CBE protein which is then toxic to the cells.

To understand why some transformants survive this possible toxic effect, we randomly selected four surviving *A. tumefaciens* single colonies and amplified the SsdA_tox_-encoding region. Sanger sequencing showed C-to-T transitions within the SsdA_tox_ domain which led to amino acid changes of D77N, S87F, G103S, and Q113stop, respectively (Fig. 1e). To validate these variants, we introduced D77N and G103S into wild-type SsdA_tox_ by site-directed mutagenesis, and assembled SsdA^D77N^-CBE and SsdA^G103S^-CBE, respectively, in level-M binary vectors. We found that both of them yielded comparable CFU to the hA3A-CBE following transformation in *E. coli* and *A. tumefaciens* (Fig. 1c, d), indicating that the toxicity of SsdA_tox_ is abolished in these variants. Previous studies have shown that SsdA_tox_ introduced high levels of random C-to-T editing in the bacterial genome when heterologously expressed in *E. coli* [32]. We could show that the toxicity of SsdA_tox_ is reduced by specific amino acid changes that possibly occur via spontaneous endogenous C-to-T editing by SsdA_tox_ itself.

### Introduction of an intron into SsdA_tox_ reduces toxicity in A. tumefaciens

**Fig. 2:**
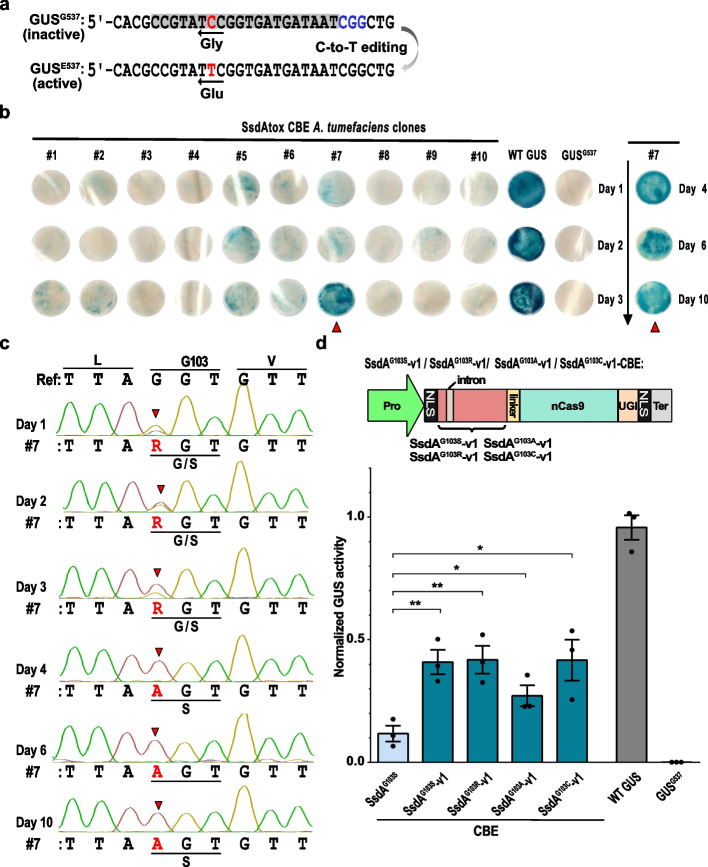
SsdA_tox_-CBE variants enable C-to-T editing of a GUS^G537^ reporter in *N. benthamiana*.** a** Schematic of the GUS^G537^ cytosine base editing reporter. The C-to-T (highlighted in red) editing in GUS^G537^ can alter the glycine codon (GGA) to a glutamic acid codon (GAA) and restore GUS activity. The protospacer is shown with gray background, and the PAM is in blue. **b** Ten different *A. tumefaciens* transformants were co-inoculated together with the GUS^537^ reporter into *N. benthamiana* leaves. Leaf disks were harvested 2 dpi then stained in GUS staining solution and de-stained in ethanol. Blue color indicates restored GUS activity. Dark blue leaf disk from clone #7 are marked by triangles. WT GUS: wild-type GUS, positive control. GUS^G537^: negative control. **c** Sanger sequencing results from *A. tumefaciens* clone #7 between days 1 and 10. Mismatches to the wild-type sequence are highlighted in red and indicated by triangles. **d** Top: Architectures of CBEs containing SsdA^G103S^-v1, SsdA^G103R^-v1, SsdA^G103A^-v1, or SsdA^G103C^-v1. An intron was introduced into the coding sequences of these SsdA variants to prohibit translation in bacteria. Bottom: C-to-T editing efficiencies of SsdA-CBE variants of the GUS^G537^ reporter. GUS activities were measured and normalized to 2 × 35S::GUS (WT GUS, positive control). Values and error bars indicate the mean ± SEM, *n* = 3. * *P* < 0.05; ** *P* < 0.01 using Student’s two-tailed unpaired *t*-test

To reduce the toxicity of SsdA in bacteria, we designed two different variants (SsdA_v1 and SsdA_v2) with an intron inserted into the coding sequence of SsdA_tox_ (Additional file 1: Fig. S2). The intron terminates translation in the absence of RNA splicing in bacteria, but allows correct translation after splicing in eukaryotes. To quantify base editing, we used a GUS-assay in *N. benthamiana*. The assay is based on an inactive GUS^G537^ allele with a missense mutation of glutamic acid (GAA) to glycine (GGA) in one of the catalytic residues. A C-to-T (G-to-A on the opposite DNA strand) conversion can revert the glycine residue to glutamic acid and restore GUS enzymatic activity [33] (Fig. 2a). We mixed the *A. tumefaciens* strains carrying SsdA_v1-CBE or SsdA_v2-CBE with a strain carrying the GUS^G537^ reporter and infiltrated *N. benthamiana* leaves. Quantification of GUS activity showed that both base editors result in base editing with SsdA_v1 being more efficient than SsdA_v2 (Additional File 1: Fig. S3). This demonstrates that SsdA can be used as the catalytic domain in a novel CBE tool.

### An engineered SsdA-CBE induces efficient C-to-T editing

To investigate whether the SsdA_tox_ variants with reduced toxicity can also function as catalytic domains in cytosine deaminases, we randomly selected 10 *A. tumefaciens* single colonies after transformation with the original SsdA_tox_-CBE binary vector. We mixed the 10 *A. tumefaciens* transformants containing SsdA_tox_-CBE individually with a strain containing the GUS^G537^ reporter, and infiltrated the mixtures into *N. benthamiana* leaves. After 48 h, leaf discs were harvested and stained for GUS activity. All 10 clones exhibited very low overall GUS activity, but some of the leaf areas showed a darker blue color suggesting that the infiltrated bacteria were a mixture of mutant variants with and without base editing activity, respectively. The 10 *A. tumefaciens* strains were further sub-cultured, and infiltrations were repeated for three consecutive days with the same *A. tumefaciens* clones. Leaf staining showed that the GUS activity of clone #7 increased from day 1 to day 3 (Fig. 2b). Sanger sequencing of clone #7 revealed a stepwise accumulation of the SsdA^G103^ variant over the wild type version (Fig. 2c).

We further sub-cultured clone #7 over 10 days and used *A. tumefaciens* suspensions from days 4, 6, and 10 for leaf infiltrations. Leaf staining showed stable GUS activity, and sequencing results showed a pure amino acid mutation of G103S in the SsdA_tox_ domain (Fig. 2b, c). These results suggest that the amino acid G103 of SsdA_tox_ plays a crucial role for the activity of the enzyme. On the one hand, the G103S mutation results in a low-toxic protein variant, and on the other hand, this amino acid change results in a highly active SsdA^G103S^-CBE.

To further investigate the relevance of this specific amino acid change of SsdA-CBE, we introduced four different types of amino acid substitutions at the G103 position (G103S, G103R, G103A, and G103C) in SsdA_v1 containing an intron in the coding sequence. These variants are named SsdA^G103S^-v1, SsdA^G103R^-v1, SsdA^G103A^-v1, and SsdA^G103C^-v1, respectively (Fig. 2d). We then tested the SsdA-CBE variants in *N. benthamiana* using the GUS^G537^ reporter. The GUS activity was normalized to a constitutive 2 × 35S::GUS construct. SsdA^G103S^-v1-CBE had approximately a fourfold increase in GUS activity compared to SsdA^G103S^ without intron.

Moreover, SsdA^G103S^-v1-CBE, SsdA^G103R^-v1-CBE, and SsdA^G103C^-v1-CBE exhibited $\sim$ 40% normalized GUS activity, while SsdA^G103A^-v1-CBE displayed $\sim$ 26% normalized GUS activity (Fig. 2d). The highly active SsdA^G103S^-v1-CBE was chosen for further analyses. Taken together, SsdA_tox_ can be engineered into an efficient cytosine base editing tool.

### SsdA^G103S^ is a ssDNA-specific deaminase

**Fig. 3:**
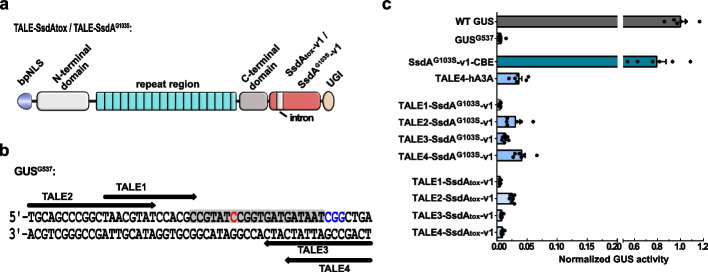
SsdA^G103S^ cannot use dsDNA as substrate.** a** Architectures of TALE-SsdA_tox_ or TALE-SsdA^G103S^ fusions. bpNLS: bipartite nuclear localization sequence; UGI: uracil glycosylase inhibitor. **b** Binding sites of four TALE arrays to the GUS^G537^ reporter. TALE binding sites are indicated by arrows in N- to C-terminal orientation. A Cas9 protospacer is in gray background, and the PAM is in blue. The target C is in red. **c** C-to-T editing efficiencies of the TALE-SsdA_tox_ and TALE-SsdA^G103S^ fusions using the GUS^537^ reporter in *N. benthamiana*. SsdA^G103S^-v1-CBE (with nCas9-UGI): positive control. GUS activities were measured and normalized to 2 × 35S::GUS (WT GUS). GUS.^537^: reporter alone (negative control). Values and error bars indicate the mean ± SEM, *n* = 6

In previous in vitro studies, purified SsdA_tox_ protein efficiently deaminated cytosines in all four sequence contexts (AC, CC, TC, and GC) using ssDNA as substrate. However, it still exhibited some residual catalytic activity toward dsDNA [32]. To investigate if SsdA can deaminate cytosine in dsDNA in vivo, we fused SsdA_tox_-v1 or SsdA^G103S^-v1 (both containing an intron within the coding sequence) to a single TALE (transcription activator–like effector) array protein (Fig. 3a). Unlike the SpCas9 nickase, the TALE array guides the deaminase to a designated dsDNA sequence without unwinding and nicking the dsDNA. Four different TALE arrays targeting the GUS^G537^ reporter were used separately. This allowed the target cytosine to be positioned at C6 or C12 downstream of the TALE-binding DNA strand (TALE1 or TALE2), or at C5 or C8 (TALE3 or TALE4) downstream of the opposite strand (Fig. 3b). When testing these fusions using the GUS^G537^ reporter in *N. benthamiana*, all the TALE-SsdA_tox_-v1 and TALE-SsdA^G103S^-v1 fusions showed background GUS activity compared to a TALE-hA3A fusion. hA3A is a ssDNA-specific deaminase and was used as a negative control. In contrast, the SsdA^G103S^-v1 fusion to nCas9-UGI (positive control) shows approximately 80% GUS activity (Fig. 3c). These results strongly suggest that the original SsdA_tox_ as well as SsdA^G103S^ are ssDNA-specific cytosine deaminases.

### Rational truncation of SsdA^G103S^-v1 to generate a very small deaminase

Crystal structure analysis revealed that SsdA contains the fundamental features of deaminase enzymes, including histidine and cysteine residues near the active site to coordinate a zinc ion, as well as three α-helices and five β-strands that make up the core fold of the enzymes in the deaminase superfamily [32].

**Fig. 4:**
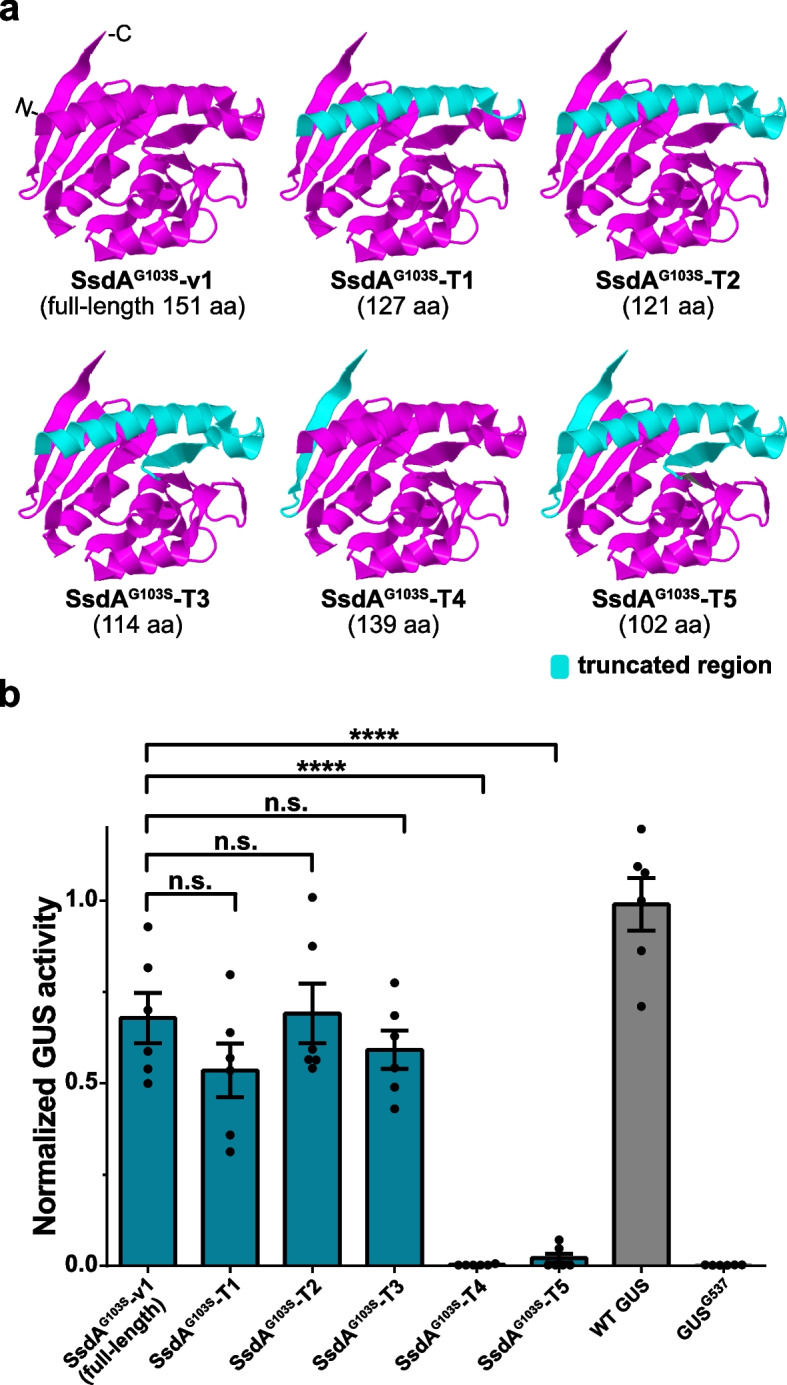
Editing activities of truncated SsdA^G103S^ variants.** a** Engineering truncations of the SsdA^G103S^ protein. Cryo-EM structure of SsdA_tox_ (PDB: 7JTU). The truncated sequences are in cyan. The final length of truncated SsdA^G103S^ proteins is listed. **b** C-to-T editing efficiencies of the truncated SsdA^G103S^ variants using the GUS^537^ reporter in *N. benthamiana*. GUS activities were measured and normalized to 2 × 35S::GUS (WT GUS). GUS^537^: reporter alone (negative control). Values and error bars indicate the mean ± SEM, *n* = 6. **** *P* < 0.0001; n.s. (not significant) using Student’s two-tailed unpaired *t*-test

We wondered whether we could rationally truncate the SsdA protein to shorten its size. We generated five differently truncated SsdA^G103S^ variants (named SsdA^G103S^-T1 to SsdA^G103S^-T5) with truncation at the N-terminal or/and C-terminal end of SsdA^G103S^ (Fig. 4a and Additional File 1: Fig. S2).

We tested these variants as CBE fusions and quantified C-to-T editing efficiency in the GUS^G537^ reporter in *N. benthamiana*. Compared to the full-length SsdA^G103S^ protein (SsdA^G103S^-v1), SsdA^G103S^-T1, SsdA^G103S^-T2, and SsdA^G103S^-T3 with truncation at the N-terminal end show comparable GUS activities, while SsdA^G103S^-T4 and SsdA^G103S^-T5 with truncation at the C- and C/N-terminal end, respectively, result only in background GUS activity (Fig. 4b). These results indicate that the β-strand located at the C-terminal end of the SsdA^G103S^ protein is crucial for the enzyme activity, and that the two α-helical and one β-strand at the N-terminal region can be truncated without reducing the enzymatic activity. Taken together, we could successfully reduce the protein size of SsdA^G103S^ to a length of only 114 amino acids.

### SsdA^G103S^-v1-CBE exhibits a narrow editing window in rice and barley protoplasts

**Fig. 5:**
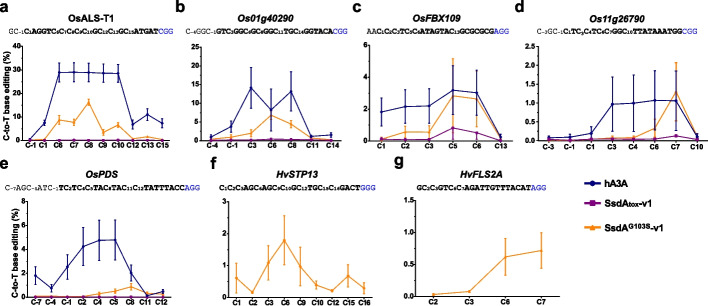
SsdA^G103S^-v1-CBE editing of genomic loci in rice and barley protoplasts.** a–e** Comparison of C-to-T editing frequencies of SsdA^G103^-v1-CBE, SsdA_tox_-v1-CBE, and hA3A-CBE at four genomic rice loci (**a**–**e**). **f**,**g** C-to-T editing frequencies of SsdA.^G103^-v1-CBE at two barley loci. The protospacer sequence is shown in bold with putative target C labelled with numbers indicating their position in the protospacer. The PAM is in blue. Error bars indicate the mean ± SEM, *n* = 3

To investigate the editing window of SsdA^G103S^-v1-CBE, we targeted five genomic loci in rice (*OsALS*, *OsPDS*, *OsFBX109*, *Os01g40290*, and *Os11g26790*) and two genomic loci in barley (*HvSTP13* and *HvFLS2A*) using protoplasts. These sites contain possible target cytosines at different positions of the protospacer. Amplicon deep sequencing results revealed that all five rice target sites were successfully edited by SsdA^G103S^-v1-CBE with varying efficiencies depending on the individual target site. In comparison to a hA3A-CBE, it had a somewhat lower activity, but also a narrower activity window at the PAM-distal protospacer (Fig. 5a–e). Specifically, the SsdA^G103S^-v1-CBE showed comparable C-to-T editing efficiency at C5 and C6 at the *OsFBX109* target site, as well as C7 at the *Os11g26790* target site when compared to the hA3A-CBE. In contrast, the SsdA_tox_-v1-CBE (wild-type SsdA_tox_ with intron in the coding sequence) showed very low to no activity at these five rice target sites, demonstrating that the G103S amino acid change particularly enhances activity. Besides from rice, SsdA^G103S^-v1-CBE exhibited efficient editing at the two barley target sites (Fig. 5f, g). Overall, our protoplast results show that the SsdA^G103S^-v1-CBE can efficiently convert C-to-T in plant genomes, with optimal target Cs ranging from C5 to C8 across the protospacer sequences.

### Efficient base editing with SsdA^G103S^-v1 in rice plants

**Fig. 6:**
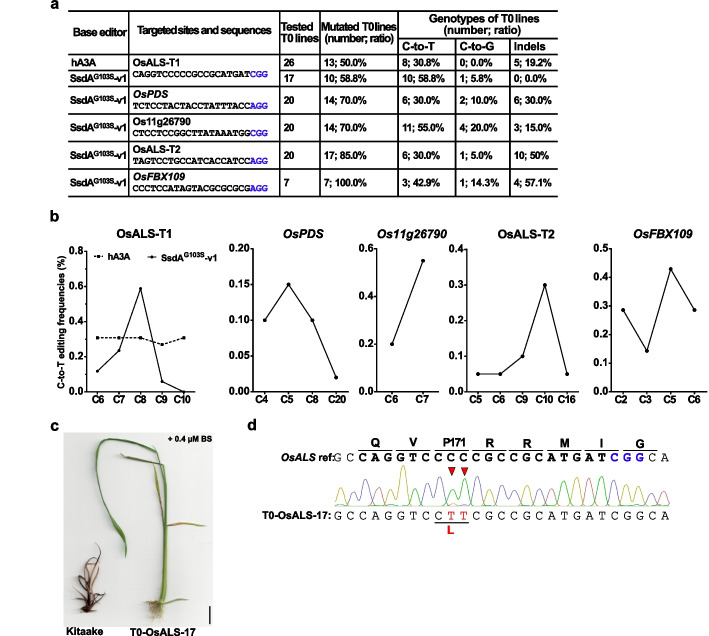
SsdA^G103S^-v1-CBE editing in rice plants.** a** Genotyping results of regenerated T0 plants transformed with hA3A-CBE or SsdA^G103S^-v1-CBE. **b** Summary of hA3A-CBE and SsdA^G103S^-v1-CBE editing outcomes at specific cytosines within the protospacers. **c** The *OsALS* P171L mutation induced by SsdA^G103S^-v1-CBE in rice confers resistance to the BS-herbicide. Bar = 1 cm. **d** Genotype of the edited T0 plant T0-OsALS-17. C-to-T conversions are indicated in red and marked with red arrows. Codons are translated in one letter code above the chromatogram for wild type sequence and the P171L mutation is shown below the chromatogram

To investigate the use of the SsdA^G103S^-v1-CBE for base editing in plants, we targeted five rice genomic loci via *Agrobacterium*-mediated transformation of rice calli. These were *OsPDS*, *Os11g26790*, and *OsFBX109*, as well as two different sites within the *OsALS* gene (OsALS-T1, OsALS-T2). Genotyping of regenerated T0 plants showed that SsdA^G103S^-v1-CBE could effectively induce C-to-T conversions at all of these target sites with an editing efficiency ranging from 30 to 58.8% (Fig. 6a and Additional file 2: Table S1). In comparison to the SsdA^G103S^-v1-CBE, we used the hA3A-CBE targeting the OsALS-T1 site. The hA3A-CBE achieved a precise C-to-T editing rate of 30.8% (8/26 plants) at the OsALS-T1 site, whereas the SsdA^G103S^-v1-CBE exhibited a higher editing efficiency of 58.8% (10/17 plants). Consistent with the protoplast results, SsdA^G103S^-v1-CBE showed a narrow editing window in these edited plants (Fig. 6b). Editing byproducts like indels and C-to-G changes were generated by both CBEs at these target sites (Fig. 6a). To investigate the heritability of the mutations that were generated by SsdA^G103S^-v1-CBE, a total of 32 individual T1 seedlings from two different T0 lines were subjected to genotyping. Sanger sequencing results showed stable inheritance of C-to-T editing in these T1 lines (Additional file 2: Table S2). Furthermore, 11 transgene-free edited rice plants were identified among these T1 lines.

The OsALS-T1 site is positioned within the rice acetolactate synthase gene (*OsALS*), specifically the region encoding the proline 171 (P171). *OsALS* confers resistance to the herbicide bispyribac-sodium (BS) when P171 is substituted with phenylalanine (F) or leucine (L) after editing of the coding sequence by CBEs [34]. To examine the BS-resistance of rice edited by SsdA^G103S^-v1-CBE, the T0 line T0-OsALS-17 was incubated on MS medium containing 0.4 µM BS (Fig. 6c). This line harbors a P171L substitution at the OsALS-T1 locus which is caused by two cytosines having been converted to thymine (Fig. 6d). Compared to wild-type Kitaake rice plants, T0-OsALS-17 exhibited a strong tolerance to BS (Fig. 6c). Taken together, we conclude that SsdA^G103S^-v1-CBEs can efficiently introduce C-to-T editing in rice and generate herbicide-tolerant rice lines.

To assess sgRNA-dependent off-target editing in the SsdA^G103S^-v1-CBE-edited rice plants, we analyzed potential off-target sites that are predicted by CRISPR-P [35]. For the OsALS-T1 site, four predicted off-target sites with 1–4 mismatches to the on-target sequence were examined (Table 1). Sequencing results from three SsdA^G103S^-v1-CBE-edited T0 plants detected off-target editing in three lines at the off-target site 1 (1 mismatch), and two out of three lines had off-target editing at the off-target site 2 (2 mismatches). No off-target editing was identified at the off-target sites 3 and 4 in two plants, respectively. At the same OsALS-T1 site, we also analyzed two hA3A-CBE-edited T0 plants and found 100% (2/2) off-target editing at both off-target site 1 and site 2, but no editing at the other two predicted off-target sites. We further tested the top predicted off-target sites at the *OsPDS*, *Os11g26790*, and OsALS-T2 target sites. The sequencing results showed that no off-target editing occurred (Additional file 2: Table S3). These results indicate that both hA3A- and SsdA^G103S^-v1-CBEs can induce off-target editing, especially at near identical sgRNA target sites with only 1–2 mismatches.

**Table 1:** Analyzing potential Cas-dependent off-target editing of hA3A-CBE and SsdA^G103S^-v1-CBE in T0 rice plants

	**Sequence**^a^	**Editor used**	**Mutation type**	**Frequency (edited plant / tested plant)**
On-target	*OsALS* (OsALS-T1) CAGGTCCCCCGCCGCATGAT**CGG**	-	-	-
Off-target site 1	*Os04g106500* CAGGTCCC*G*CGCCGCATGAT**CGG**	hA3A	Indels	100% (2/2)
		SsdA^G103S^-v1	Indels & C-to-T	100% (3/3)
Off-target site 2	*Os04g106900* CAGG*C*CCC*G*CGCCGCATGAT**CGG**	hA3A	C-to-T	100% (2/2)
		SsdA^G103S^-v1	C-to-T	66.7% (2/3)
Off-target site 3	*Os05g247500* C*C*GGT*T*C*G*CCGCC*T*CATGAT**TGG**	hA3A	WT	0% (2/2)
		SsdA^G103S^-v1	WT	0% (2/2)
Off-target site 4	*Os08g020200* CAG*A*TCC*G*CCGCCGCA*C*G*C*T**CGG**	hA3A	WT	0% (2/2)
		SsdA^G103S^-v1	WT	0% (2/2)

^a^: Mismatched sequences in the protospacer are in italics, and the PAMs are in bold

### SsdA^G103S^-v1 base editing activity in mammalian cells

**Fig. 7:**
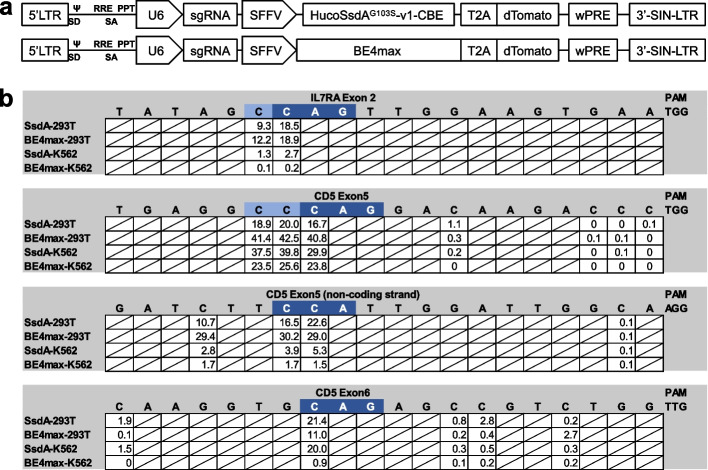
Lentiviral vector design and SsdA^G103S^-v1-CBE activity in mammalian cells.** a** The human codon-optimized SsdA^G103S^-v1-CBE (HucoSsdA^G103S^-v1-CBE) or BE4max-CBE were cloned into a self-inactivating lentiviral vector (SIN-LV) and expressed under a spleen focus-forming virus (SFFV) promoter. A ribosomal skipping sequence (T2A) was fused to the C-terminus of both CBEs to co-express a dTomato fluorescent protein. The sgRNA is expressed under a human U6 promoter. **b** A summary of both CBEs used in human 293 T and K562 cells, including the targeting sequence, along with the frequency of C-to-T conversions. Sequences in dark blue represent bases where C-to-T conversions lead to de novo stop codon formation. LTR, long terminal repeat; Ψ, packaging element; RRE, Rev response elements; PPT, polypurine tract; SD, splice donor; SA, splice acceptor; wPRE, Woodchuck hepatitis virus posttranscriptional regulatory element

To determine if SsdA^G103S^-v1-CBE is active in mammalian cells, we cloned a human codon-optimized SsdA^G103S^-v1-CBE (HucoSsdA^G103S^-v1-CBE) into an all-in-one lentiviral vector (LV; Fig. 7a). sgRNAs were selected to target two genes for knockout by generating premature stop codons in the transcripts: IL7RA on exon 2 and CD5 on exons 5 and 6. These sgRNAs were cloned into the HucoSsdA^G103S^-v1-CBE and BE4max all-in-one LVs for comparison. The LVs were packaged using 293 T cells, and the viral supernatant was used to transduce K562 cells. Genomic DNA was then extracted from both the transfected 293 T cells and the LV-transduced K562 cells.

In both the LV plasmid transfected 293 T cells and LV-transduced K562 cells, we observed 3 to 48% total mutation in the targeted regions, including C-to-T conversions that resulted in de novo stop codons in all targets edited with either CBE (Fig. 7b). We also observed a low level of indels within the target windows generated by both CBEs.

Our findings confirm that the newly developed SsdA^G103S^-v1-CBE is active in both mammalian and plant cells, highlighting its potential applications in crop improvement and clinical settings.

### A structure-based phylogeny of SsdA_tox_ homologs

**Fig. 8:**
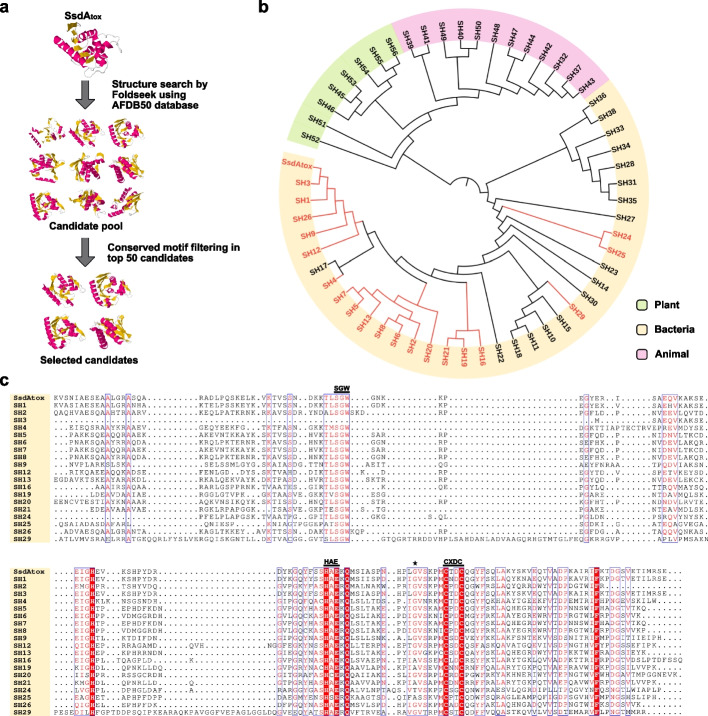
Identifying structurally related proteins of SsdA_tox_ using Foldseek.** a** Workflow of the SsdA_tox_ structure search based on Foldseek and filtering for conserved motifs. **b** Phylogenetic clustering of the top 50 highest-scored candidates and six manually selected candidates from plants. Eighteen candidates labelled in red contain the conserved motifs from SsdA_tox_. **c** Sequence alignment of the 18 candidates to SsdA_tox_, with conserved motifs shown above the alignment. Amino acid G103 from SsdA_tox_ is indicated by an asterisk

The common methodology for annotating and analyzing proteins relies on a one-dimensional (1D) amino acid similarity search, which is unable to fully reveal the functional characteristics of proteins [36, 37]. Comparing protein structures using three-dimensional (3D) superposition is more sensitive in identifying distantly related proteins with common structural or enzymatic functions^31,38^. Here, we used the protein structure search tool Foldseek [38] for mining proteins that might be functionally related to SsdA_tox_ (Fig. 8a). The structure of SsdA_tox_ was aligned to the AlphaFold Protein Structure Database (AFDB50) [39] and the top 50 highest-scored candidates (SsdA_tox_ Homolog, SH1 to SH50) as well as six manually selected related proteins from plants (SH51 to SH56) were used for phylogenetic clustering (Fig. 8b). We further scanned these candidates for conserved motifs that are a hallmark of the BaDTF2 (SsdA_tox_) deaminase subfamily and differentiate it from the mRNA-targeting members of the DYW-family [32]. A HAE motif is believed to contain a catalytic glutamate and its histidine likely coordinates together with two cysteines of a CxDC motif a Zn^2+^ ion. In addition, a Ser-Gly-Trp (SGW) motif lies in a loop region in close proximity to the active site of SsdA_tox_. All three motifs were identified in 18 of the 50 candidates (Fig. 8c). We speculate that these 18 candidates, which exhibit structural similarity to SsdA_tox_, may possess cytosine deaminase catalytic activities and could possibly also be engineered into CBEs.

## Discussion

Members of the cytosine deaminase superfamily in prokaryotes and eukaryotes catalyze base deamination in a wide variety of contexts [31, 40]. Deaminases that have been incorporated into genome editing tools either use DNA as a substrate (ssDNA [8] or dsDNA [22]) or have been artificially evolved to accept DNA instead of RNA [9]. In mammalian systems, rat APOBEC1 and derivatives are commonly used in CBEs [8], but human APOBEC3A and APOBEC3B are significantly more active [41, 42], which results in a dramatic difference for plants [17, 43].

Here, we have developed a novel CBE, which is highly active in plants. For this, we developed an engineered variant of the bacterial SsdA_tox_ deaminase from *P. syringae*. SsdA_tox_ is one of the interspecies bacterial toxin system-dependent deaminases and is classified into the DYW family whose evolutionary separation from all other currently used enzymes in CBEs predates the origin of eukaryotes [32, 40]. Plant and fungal enzymes from this family are often fused to PPR or ankyrin repeats and contribute to editing of organellar mRNA transcripts [44, 45]. Accordingly, synthetic PPR-DYW proteins have been used for targeted base editing of RNA [46]. In contrast, for SsdA_tox_, DNA and not RNA is the only physiological substrate with a very high preference for ssDNA over dsDNA [32], which is desired for its use in a CBE. Bacterial type VI secreted toxins are often fused to additional domains like PAAR or Rhs to facilitate their assembly at the tip of a type VI secretion system (Additional file 1: Fig. S1) [47–49]. For our approach, we truncated SsdA_tox_ to the predicted catalytic domain (151 aa).

Expression of free SsdA_tox_ is toxic and resulted in numerous C•G-to-T•A transitions in *E. coli* [32], but we reasoned that the gene should be tolerated when placed under control of a plant-specific promoter and fused to nCas9, which guides it to specific genomic locations and produces a local ssDNA substrate. This was the case for our clonings in *E. coli*, but we experienced a significant toxicity in *A. tumefaciens*, which possibly resulted from a low level of background expression by the plant 2 × 35S-promoter in *A. tumefaciens*. On the other hand, this negative selective pressure allowed us to isolate surviving *A. tumefaciens* colonies that contained suppressor mutations in SsdA_tox_ resulting in amino acid changes or stop codons. Conspicuously, the isolated mutations are predominantly C-to-T transitions, rendering it possible that the mutagenic activity of SsdA_tox_ itself caused them. From these transformants, we readily identified clones with high in planta editing activity. The SsdA^G103S^ variant and others with amino acid exchanges of G103 showed no toxicity while enabling efficient C-to-T conversion. This is counterintuitive because mutations with low or abolished deaminase activity should have a selective advantage if this enzyme is causing the toxicity. The critical residue (G103) lies in the 3rd β-strand in relative proximity of a postulated catalytic glutamate residue. Structural data suggest that residue G103 of SsdA_tox_ interacts with residues S34 and S35, while in SsdA^G103S^, the larger side chain of S103 interacts with four residues: S34, S35, V104, and A130 (Additional file 1: Fig. S4). The structure of SsdA_tox_ exhibits an anti-parallel arrangement of beta-strands 4 and 5, whereas in the APOBEC family, these β-strands are parallel [32]. We speculate that the interaction between residue S103 and A130 in β-strand 4 might affect the activity pocket of the enzyme, thereby restricting the activity of SsdA. This could result in lower toxicity toward untargeted regions and a more specific affinity to the target R-loop region generated by nCas9.

In a parallel study, a non-peer-reviewed preprint described SsdA as a deaminase in CBEs for genome editing in mammalian cells [50]. In that report, three missense mutations (P282S, Y335R, K392E) within SsdA enhance cytosine base editing efficiency, but they are located at different positions to G103 identified by us (which would correspond to G362 in [50]). Future studies will reveal how such mutations influence the activity of DYW proteins.

We further exploited the use of introns to reduce toxicity by blocking translation of the toxic SsdA_tox_ in bacteria, which was successful. Interestingly, we found that an intron-containing SsdA^G103S^ variant (SsdA^G103S^-v1) shows also significantly higher C-to-T editing efficiency than SsdA^G103S^ lacking the intron (SsdA^G103S^) in planta. Beneficial effects of introns to expression efficiencies of transgenes are known and are possibly due to increased export from the nucleus to the cytoplasm in plant cells [42]. Accordingly, we propose SsdA^G103S^-v1-CBE as the preferred tool for plants.

CBEs exhibit different editing windows. In plants, APOBEC1 (nCas9-PBE) shows an editing window from C3 to C9 within the protospacer [17], whereas hA3A (A3A-PBE) has a broader editing window spanning from C1 to C17 [18]. In this study, we demonstrate that the new SsdA^G103S^-v1-CBE prefers a narrower editing window of C5 to C8, which is beneficial if a specific target site shall be edited, but multiple cytosines are present within the protospacer. For example, hA3A edited five Cs (C6 to C10) simultaneously at the OsALS-T1 site, while the SsdA^G103S^-v1-CBE preferred to edit C7 and C8 (Fig. 6b and Additional file 2: Table S1). A change from C7 and/or C8 to T results in Pro171 (CCC) being altered to Phe (TTC), Leu (CTC), or Ser (TCC), which confers resistance to the BS-herbicide in rice [34].

CBEs can induce undesired off-target base substitutions [51, 52]. In this study, we found Cas-dependent off-target editing induced by hA3A-CBE or SsdA^G103S^-v1-CBE in T0 rice plants. To address the Cas-dependent off-targets, high-fidelity Cas9 variants can be incorporated into the CBE architectures to replace the wild-type nickase Cas9 [53–58]. Cas-independent off-target effects of SsdA^G103S^-v1-CBE were not analyzed in this study. Considering the comparable Cas-dependent off-target editing rates of hA3A-CBE and SsdA^G103S^-v1-CBE, and our efficiency of achieving fully edited plants with no apparent toxicity using either editing tool, we estimate that both CBEs possible cause similar off-target rates overall.

In addition to plant cells, we have also shown that our SsdA^G103S^-v1-CBE is functional in mammalian cells, as tested on two human cell lines: the 293T epithelial cell line and K562, a myeloid leukemia cell line, with comparable efficiency to BE4max.

Cytosine deamination by deaminase enzymes can be sequence context-specific. For example, APOBEC1 and APOBEC3 show preferential deamination of cytosines in a 5´-TC sequence context [8, 59]. According to structural studies, hA3A binds ssDNA substrates in a U-shaped conformation, with the target cytosine inserted deep into the zinc-coordinating active site pocket, while the − 1 thymine base flipped out and fits into a groove between flexible loops, making direct hydrogen bonds with the protein [42]. Previous in vitro studies indicated that SsdA_tox_ enables the deamination of cytosines in all 5´-NC sequence contexts with a slight preference for neighboring pyrimidines [32]. Our editing results also show that SsdA^G103S^-v1 has a flexible substrate sequence context in plants. This suggests that SsdA^G103S^-v1 can edit all cytosines within the editing windows without a sequence context restriction.

The SsdA_tox_ (151 aa) we used in this study is already smaller than currently used cytosine deaminases, such as APOBEC1 (227 aa) [8], hA3A (198 aa) [41], AID (182 aa) [8], PmCDA1 (207 aa) [60], TadA8e variants (166 aa) [14–16, 61], and mini-Ssd7 (158 aa) [31]. Moreover, we could further truncate SsdA^G103S^ to a length of 114 aa without sacrificing activity, making SsdA^G103S^-derived base editors a promising candidate for delivery into cells using size-limited methods (e.g., adeno-associated virus). Using a state-of-the-art structure search algorithm, we discovered a group of related proteins that could potentially function as deaminases and are candidates for additional future cytosine base editor systems.

## Conclusions

In summary, the new SsdA^G103S^-derived base editors expand the genome editing toolbox and can precisely and efficiently target C-to-T conversion in plants and mammalian cells. They exhibit comparable activity to highly optimized deaminases in established CBEs while providing a narrower editing window which allows more precise base targeting. The truncated SsdA^G103S^-T3 is with 114 aa length by far the smallest deaminase in a genome editing tool to date. Taken together, SsdA^G103S^-derived CBEs provide valuable alternatives for crop improvement and human gene therapy.

## Methods

### Plasmid construction

The SsdA_tox_ was amplified from *Pseudomonas syringae* pv. *aptata* GSPB1067. SsdA variants were generated by point mutation PCR. All plasmids used in this work were assembled based on Modular Cloning-compatible (MoClo) vectors (Additional file 2: Table S4 and Additional file 1: Supplementary sequences) [62–64]. The 2 × 35S promoter was used to express CBEs in the leaf infiltration and protoplast assays. The ZmUbi promoter was used to express SsdA^G103S^-v1-CBE in rice transformation. All the components were subcloned in individual modules that can be assembled using Golden-Gate Cloning [65]. Oligos used in this study are listed in Additional file 2: Table S4. In addition, a human codon-optimized SsdA^G103S^-v1-CBE (HucoSsdA^G103S^-v1-CBE) was cloned into an all-in-one self-inactivating (SIN) lentiviral vector (LV) for gene transfer into mammalian cells. The SIN-LV contains a U6 promoter to express the sgRNA and uses a spleen focus-forming virus (SFFV) promoter to express the HucoSsdA^G103S^-v1-CBE and dTomato fluorescent protein, linked by a T2A ribosomal skipping sequence. sgRNA targeting human IL7RA and CD5 were cloned into the all-in-one SIN-LV with HucoSsdA^G103S^-v1-CBE or BE4Max [66].

### Lentiviral vector production and transduction

Lentiviral vectors were generated as previously described [67, 68]. Briefly, subconfluent 293 T cells were cultured in DMEM supplemented with 10% fetal calf serum, 1% sodium pyruvate, and penicillin/streptomycin and transfected using the calcium phosphate method. The all-in-one HucoSsdA^G103S^-v1-CBE lentiviral vector plasmids were co-transfected with the helper plasmids HIV-Rev, HIV-gag/pol, and VSV-g glycoprotein. Supernatants were collected at 36 and 48 h post-transfection and filtered through 0.22-μm filters. The supernatant was then added to K562 cells for transduction in the presence of 1 mg/ml Synperonic® F 108 (Sigma-Aldrich) [69], and incubated for 48 h before genomic DNA extraction.

### Nicotiana benthamiana infiltration and GUS reporter assay

*N. benthamiana* plants were grown in a greenhouse with 16 h of light, a relative humidity of 40–60%, and temperatures of 23 °C/19 °C during the day/night, respectively. Four- to six-week-old plants were used for *A. tumefaciens* inoculation experiments. GUS reporter assays were performed as previously described [70]. Briefly, *A. tumefaciens* GV3101 strains containing a CBE construct and the GUS^G537^ reporter construct, respectively, were mixed 1:1 with an OD_600_ of 0.8 and inoculated into *N. benthamiana* leaves. After 2 days, two leaf discs (diameter 0.8 cm) were harvested from the inoculation spots. For leaf staining, leaf disks were stained in X-Gluc solution and de-stained in ethanol. For qualitative GUS assays, leaf tissues were homogenized and incubated with 4-methyl-umbelliferyl-β-D-glucuronide. GUS activities were measured using a TECAN reader (360 nm excitation and 465 nm emission). Proteins were quantified by NanoDrop™ One (Thermo Fisher Scientific).

### Protoplast isolation and transformation

Two-week-old leaves from rice cultivar Kitaake or barley cultivar Golden Promise were used to prepare protoplasts. Rice and barley protoplast isolation and transformation were performed as previously described [71]. Twenty micrograms plasmid DNA per construct were introduced into protoplasts by PEG-mediated transfection. The transfected protoplasts were incubated at room temperature. After 48 h, the protoplasts were collected and the genomic DNA extracted.

### Rice stable transformation

Rice cultivar Kitaake was used for genetic transformation *in this study*, as previously described [72]. Briefly, *A. tumefaciens* strains EHA105, containing SsdA^G103S^-v1-CBE and sgRNA, as well as a *hygromycin* resistance gene as a selection marker, were used to transform calli. Then the transformed calli were transferred to selection plates containing 50 mg/l hygromycin. Regenerated calli were moved to rooting medium, then subjected to genotyping (Additional file 2: Table S1). For herbicide-resistance assay, 3-week-old regenerated seedlings were cultured on MS medium containing 0.4 µM bispyribac-sodium, and grown at a temperature of 28 °C under a photoperiod of 16 h light and 8 h dark per day.

### DNA extraction and identification of mutants

We used the innuPREP Plant DNA Kit (Analytik Jena) to extract genomic DNA from regenerated plants and protoplasts DNA. The targeted sequences were amplified with site-specific primers (Additional file 2: Table S5). The PCR products were purified with the GeneJET Gel Extraction Kit (Thermo Fisher Scientific) and sequenced by Sanger sequencing (plants) or amplicon deep sequencing (protoplasts). Off-target sites were predicted by the online tool CRISPR-P [32]. Based on the off-target score, the top three or four predicted off-target sites of the used sgRNAs were selected as potential off-target sites (Table 1 and Additional file 2: Table S3). Site-specific primers (Additional file 2: Table S5) were used to amplify the potential off-target sites and T-DNA. The PCR products were purified and Sanger sequenced.

### Amplicon deep sequencing and data analysis

PCR amplicons were purified with the GeneJET Gel Extraction Kit (Thermo Fisher Scientific) or QIAquick Gel Extraction Kit (Qiagen), then quantified using a Qubit™ 1X dsDNA High Sensitivity Kit or NanoDrop (Thermo Fisher Scientific). Equal amounts of PCR products were pooled and sequenced (GENEWIZ, AMPLICON-EZ). Amplicon deep sequencing was performed three times for each target location using genomic DNA isolated from three different protoplast transformation experiments. For mammalian cells, deep sequencing for each targeted location was performed once from two different cell types. The target sites in the sequenced reads were analyzed for mutations using CRISPResso2 [73] (crispresso2.pinellolab.org).

### Identifying structurally related proteins of SsdA_tox_

The structure of SsdA (PDB: 7JTU) was used for a structure search using the Foldseek webserver (https://search.foldseek.com) [38]. Top 50 candidates from the AFDB50 database [39] were selected for conserved motif filtering. Details of the identified candidate proteins are listed in Additional file 2: Table S6. Sequences were aligned using ClustalW and displayed by ESPript 3.0 [74]. The SsdA homologs were used to construct a phylogenetic tree using Geneious Prime (version 2019) Tree Builder (default parameters), and visualized in ITOL (https://itol.embl.de/).

### Statistical analysis

All values are shown as mean ± SEM (standard error of the mean). Statistical differences between the values were tested using two-tailed unpaired Student’s *t* tests by GraphPad (Prism; www.graphpad.com). No other scripts and software were used than those mentioned in the “ Methods” section.

## Peer review information

Wenjing She was the primary editor of this article and managed its editorial process and peer review in collaboration with the rest of the editorial team. The peer review history is available in the online version of this article.

## Supplementary Information

Additional file 1: Supplementary figures S1-S4. Fig. S1. SsdA domain structure, function, and phylogenetic family. Fig. S2. Schematic diagrams of cytosine base editors. Fig. S3. C-to-T editing efficiencies of the intronized SsdA-CBEs and hA3A-CBE in N. benthamiana. Fig. S4. Cryo-EM structure of SsdAtox and AlphaFold2-predicted structure of SsdAG103S. Supplementary Sequences. Sequences of cytosine base editor architectures.

Additional file 2: Supplementary tables S1-S6. Table S1. Genotyping results of regenerated T0 plants transformed with SsdAG103S-v1-CBE or hA3A-CBE. Table S2. Inheritance of mutations in rice T1 lines. Table S3. Analyzing potential Cas-dependent off-target editing of SsdAG103S-v1-CBE in T0 plants. Table S4. Plasmids used in this study. Table S5. Oligos used in this study. Table S6. Top 50 candidates from the AlphaFold Protein Structure Database (AFDB50) with structural similarity to SsdAtox.

## Data Availability

The amplicon sequencing data have been deposited in the NCBI BioProject database: PRJNA1087292 [75] and PRJNA1205495 [76].
