# Supplementary material for: Engineering a bacterial toxin deaminase from the DYW-family into a novel cytosine base editor for plants and mammalian cells
Source: Genome Biol. 2025 Feb 3;26:18. doi: 10.1186/s13059-025-03478-w (PMC11789416; doi:10.1186/s13059-025-03478-w)
Supplement: Supplementary file 1 — Additional file 1: Supplementary figures S1-S4. Fig. S1. SsdA domain structure, function, and phylogenetic family. Fig. S2. Schematic diagrams of cytosine base editors. Fig. S3. C-to-T editing efficiencies of the intronized SsdA-CBEs and hA3A-CBE in N. benthamiana. Fig. S4. Cryo-EM structure of SsdAtox and AlphaFold2-predicted structure of SsdAG103S. Supplementary Sequences. Sequences of cytosine base editor architectures. [file 13059_2025_3478_MOESM1_ESM.pdf]

Engineering a bacterial toxin deaminase from the DYW-family into a novel cytosine base editor for plants and mammalian cells

Dingbo Zhang[1,2], Fiona Parth[1], Laura Matos da Silva[1], Teng-Cheong Ha[3,4], Axel Schambach[3,4,5] and Jens Boch[1,*]

[1]Institute of Plant Genetics, Leibniz Universität Hannover, Herrenhäuser Str. 2, 30419 Hannover, Germany

[2]Research Institute of Biology and Agriculture, University of Science and Technology Beijing, Beijing 100083, China

[3]Institute of Experimental Hematology, Hannover Medical School, Hannover, Germany

[4]REBIRTH - Research Center for Translational Regenerative Medicine, Hannover Medical School, Hannover, Germany

[5]Division of Hematology/Oncology, Boston Children's Hospital, Harvard Medical School, Boston, MA, USA

*Corresponding author: JB, jens.boch@genetik.uni-hannover.de

Fig. S1. SsdA domain structure, function, and phylogenetic family................ 2

Fig. S2. Schematic diagrams of cytosine base editors.................................. 3

Fig. S3. C-to-T editing efficiencies of the intronized SsdA-CBEs and hA3A-CBE in *N. benthamiana*... 4

Fig. S4. Cryo-EM structure of SsdAtox and AlphaFold2-predicted structure of SsdA^{G103S}. 5

Supplementary Sequences. Sequences of cytosine base editor architectures.. 6

a

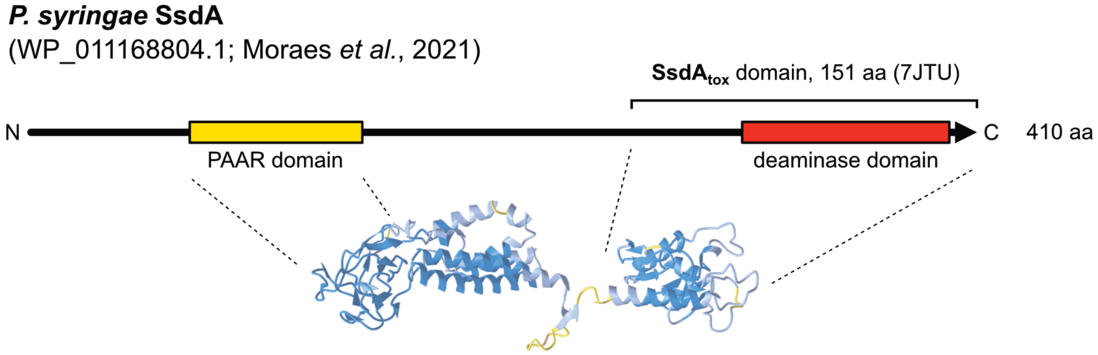

b

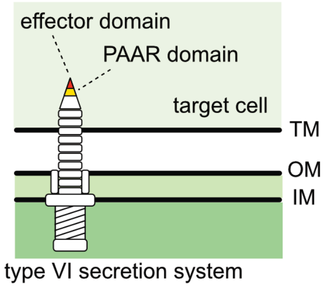

c

% identity of $SsdA_{tox}$ domains

	Pap	*Pph*	*Ppi*	*Psy*
***P. syringae* pv. *aptata* (*Pap*)**				
***P. syringae* pv. *phaseolicola* (*Pph*)**	99.3			
***P. syringae* pv. *pisi* (*Ppi*)**	99.3	98.7		
***P. syringae* pv. *syringae* (*Psy*)**	100.0	99.3	99.3	
***P. syringae* WP_011168804 (WP)**	99.3	100.0	98.7	99.3

Fig. S1. SsdA domain structure, function, and phylogenetic family.
(a) Protein domains of full-length SsdA and its predicted AlphaFold structure. **(b)** SsdA is a predicted type VI-secreted toxin. PAAR domains are typical linkers to attach toxin proteins to the tip of a type VI secretion system (T6SS). The T6SS spans both bacterial membranes and punctures other bacterial cells upon contact to release the toxin into the cytoplasm of the target cell. **(c)** SsdA was originally described from *Pseudomonas syringae* and highly identical proteins are found in many different pathovars of these plant-pathogenic bacteria.

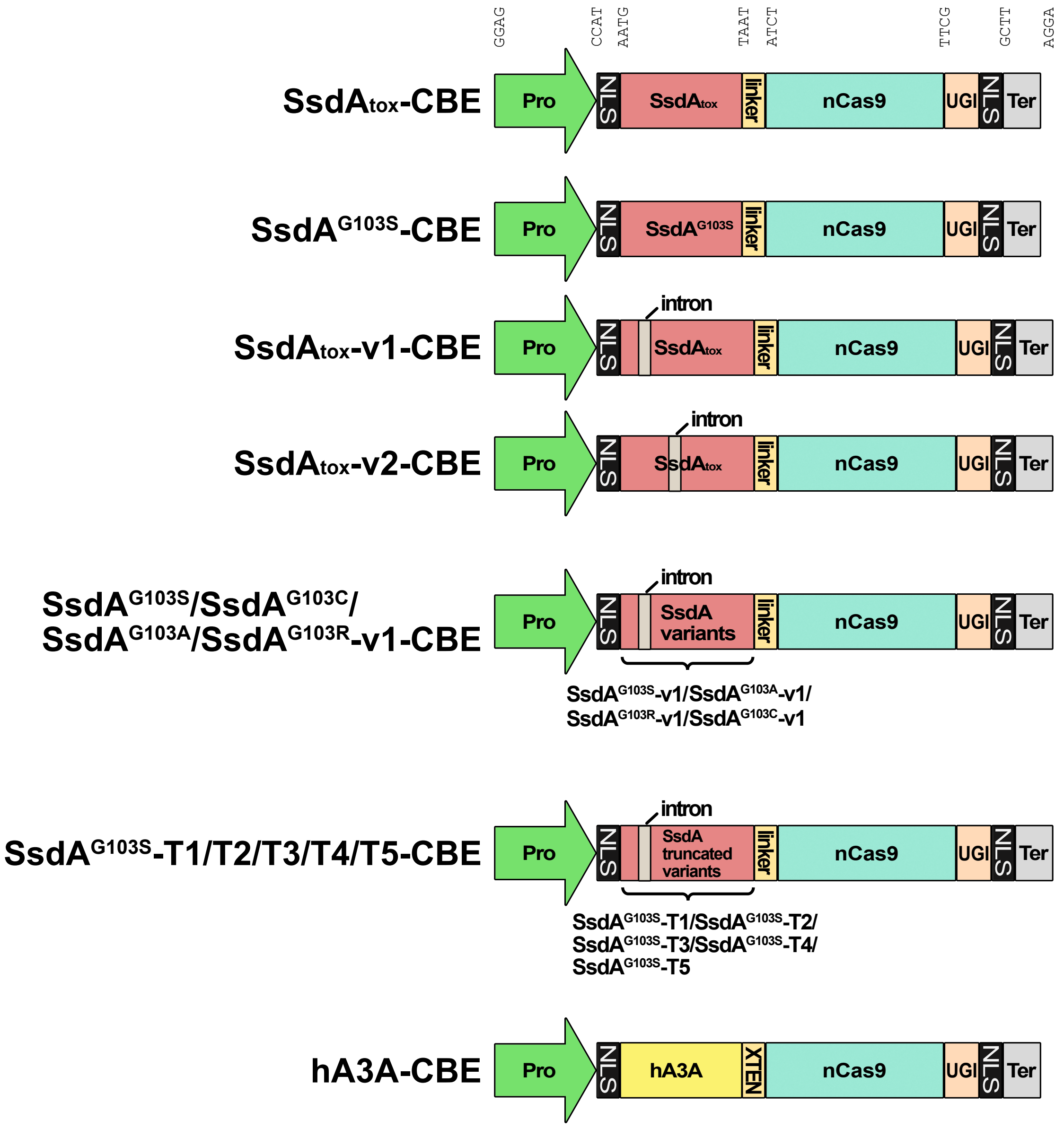

Fig. S2. Schematic diagrams of cytosine base editors.

Pro: promoter. NLS: nuclear localization signals. Linker: 32 aa-linker. XTEN: XTEN linker. hA3A: human APOBEC3A. nCas9: nickase SpCas9 (D10A). UGI: uracil DNA glycosylase inhibitor. Ter: terminator. Four-base overhang sequences are shown on the modules.

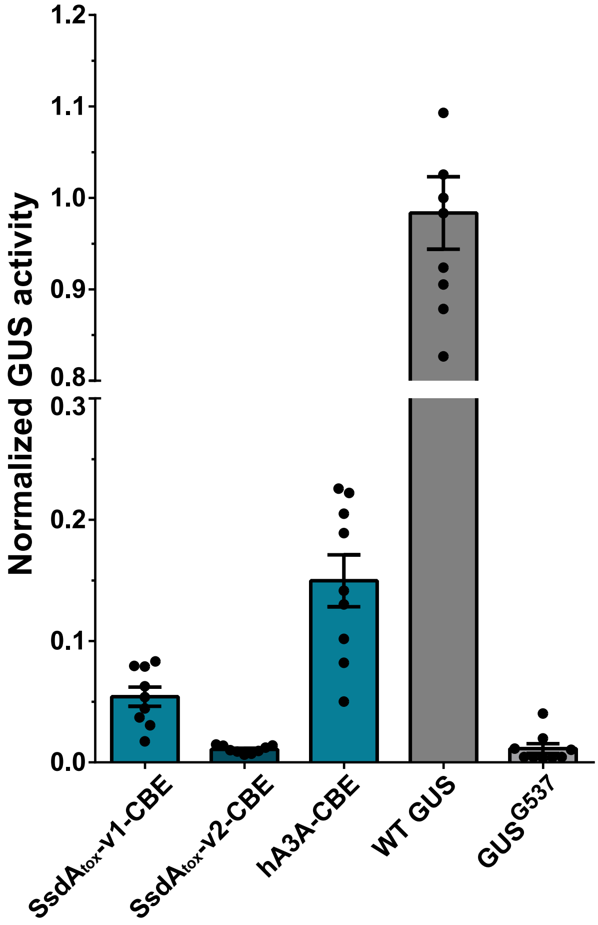

Fig. S3. C-to-T editing efficiencies of the intronized SsdA-CBEs and hA3A-CBE in *N. benthamiana*.

A. tumefaciens strains containing CBE or GUS^{G537} reporter were mixed and infiltrated into *N. benthamiana* leaves. GUS activities were measured and normalized to 35S::GUS (WT GUS, positive control). Values are confirmed in independent experiments. Values and error bars indicate the mean ± SEM, n = 9.

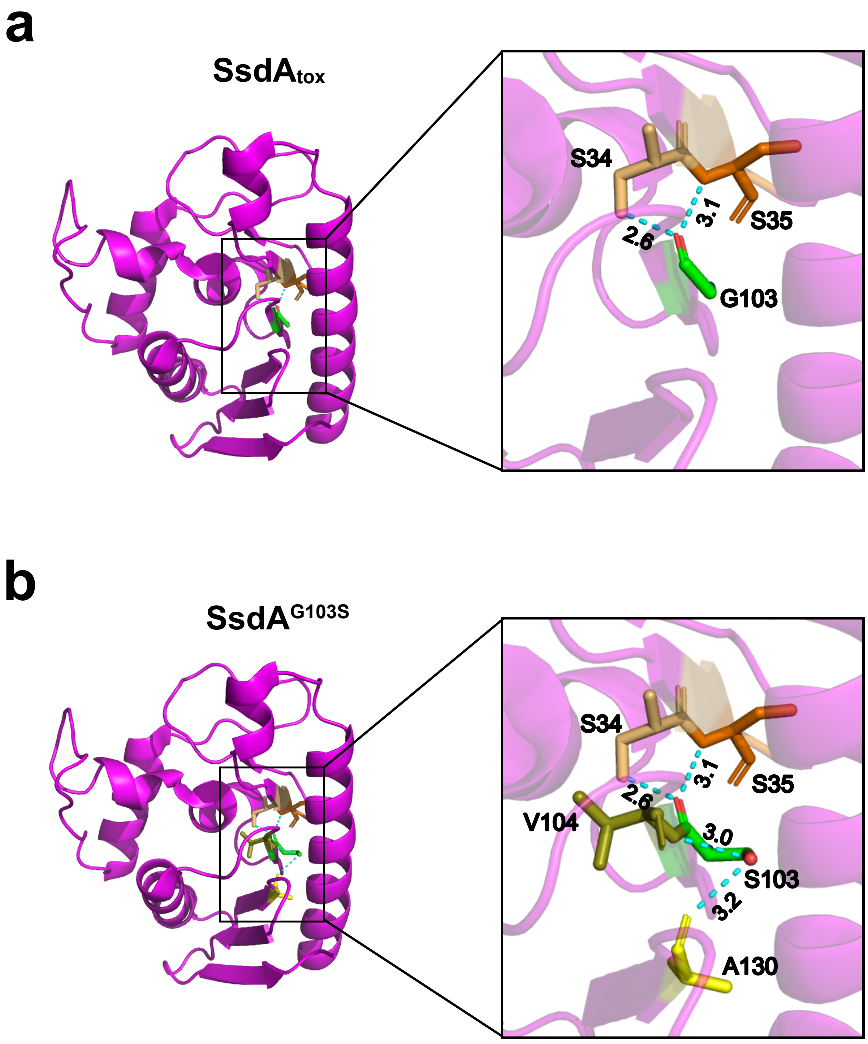

Fig. S4. Cryo-EM structure of SsdA$_{tox}$ and AlphaFold2-predicted structure of SsdA^{G103S}.
(a) Cryo-EM structure of SsdA$_{tox}$ (PDB: 7JTU). Residues S34 and S35 interact with G103, as indicated by cyan lines and numbers. **(b)** AlphaFold2-predicted structure of the SsdA$_{tox}$ variant SsdA^{G103S}. Residues (S34, S35, V104, and A130) interacting with S103 are indicated with cyan lines and numbers.

Supplementary Sequences. Sequences of cytosine base editor architectures.

General architecture of SsdA-CBEs (from N- to C-terminus):

bpNLS-deaminase-linker-nCas9-UGI-*NLS*

SsdA$_{tox}$:

AAGGTGTCAAATATTGCGGAGTCCGAAGCTGCGCTTGGTCGTGCATCACAGGCCAGAGCCGACCTTCCG
CAAAGTAAAGAGTTGAAGGTAAAAACTGTTTCATCTAATGATAAGAAAACATTAAGTGGTTGGGGTAAT
AAAAAGCCCGAAGGTTATGAGAGGATTTCTGCGGAGCAGGTCAAGGCTAAGTCTGAAGAAATTGGGCAT
GAGGTGAAATCACACCCATACGACAGAGATTACAAAGGGCAGTACTTTTCATCTCATGCAGAGAAACAG
ATGTCAATCGCAAGCCCAAATCATCCTTTAGGTGTTTCGAAGCCTATGTGTACTGATTGTCAGGGGTAC
TTTTCTCAGCTGGCTAAGTATAGTAAGGTTGAGCAGACAGTAGCAGATCCTAAAGCGATACGTATTTTT
AAGACCGATGGTTCTGTCGAAACAATTATGAGGTCGGAA

SsdA$_{tox}$ (Amino acid sequence, 151 aa):

KVSNIAESEAALGRASQARADLPQSKELKVKTVSSNDKKTLSGWGNKKPEGYERISAEQVKAKSEEIGH
EVKSHPYDRDYKGQYFSSHAEKQMSIASPNHPLGVSKPMCTDCQGYFSQLAKYSKVEQTVADPKAIRIF
KTDGSVETIMRSE

SsdA_v1 (Intron is underlined.):

AAGGTGTCAAATATTGCGGAGTCCGAAGCTGCGCTTGGTCGTGCATCACAGGCCAGAGCCGACCTTCCG
CAAAGTAAAGAGTTGAAGGTAAAAACTGTTTCATCTAATGATAAGAAAACATTAAGTGGTTGGGGTAAT
AAAAAGCCCGAAGGTAAGTCTTACTCTCTCTTTTTTGGTCTGTATTTTTAATTTTTTGAAGTATACTAT
TTGTACTGACGCTAATAATCTTTTTTCGCAGGTTATGAGAGGATTTCTGCGGAGCAGGTCAAGGCTAAG
TCTGAAGAAATTGGGCATGAGGTGAAATCACACCCATACGACAGAGATTACAAAGGGCAGTACTTTTCA
TCTCATGCAGAGAAACAGATGTCAATCGCAAGCCCAAATCATCCTTTAGGTGTTTCGAAGCCTATGTGT
ACTGATTGTCAGGGGTACTTTTCTCAGCTGGCTAAGTATAGTAAGGTTGAGCAGACAGTAGCAGATCCT
AAAGCGATACGTATTTTTAAGACCGATGGTTCTGTCGAAACAATTATGAGGTCGGAA

SsdA_v2 (Intron is underlined.):

AAGGTGTCAAATATTGCGGAGTCCGAAGCTGCGCTTGGTCGTGCATCACAGGCCAGAGCCGACCTTCCG
CAAAGTAAAGAGTTGAAGGTAAAAACTGTTTCATCTAATGATAAGAAAACATTAAGTGGTTGGGGTAAT
AAAAAGCCCGAAGGTTATGAGAGGATTTCTGCGGAGCAGGTCAAGGCTAAGTCTGAAGAAATTGGGCAT
GAGGTAAGTCTTACTCTCTCTTTTTTGGTCTGTATTTTTAATTTTTTGAAGTATACTATTTGTACTGAC
GCTAATAATCTTTTTTCGCAGGTGAAATCACACCCATACGACAGAGATTACAAAGGGCAGTACTTTTCA
TCTCATGCAGAGAAACAGATGTCAATCGCAAGCCCAAATCATCCTTTAGGTGTTTCGAAGCCTATGTGT
ACTGATTGTCAGGGGTACTTTTCTCAGCTGGCTAAGTATAGTAAGGTTGAGCAGACAGTAGCAGATCCT
AAAGCGATACGTATTTTTAAGACCGATGGTTCTGTCGAAACAATTATGAGGTCGGAA

SsdA^{D77N} (Amino acid sequence):

KVSNIAESEAALGRASQARADLPQSKELKVKTVSSNDKKTLSGWGNKKPEGYERISAEQVKAKSEEIGH
EVKSHPYNRDYKGQYFSSHAEKQMSIASPNHPLGVSKPMCTDCQGYFSQLAKYSKVEQTVADPKAIRIF
KTDGSVETIMRSE

SsdA^{G103S}_v1 (Amino acid sequence):

KVSNIAESEAALGRASQARADLPQSKELKVKTVSSNDKKTLSGWGNKKPEGYERISAEQVKAKSEEIGH
EVKSHPYDRDYKGQYFSSHAEKQMSIASPNHPLSVSKPMCTDCQGYFSQLAKYSKVEQTVADPKAIRIF
KTDGSVETIMRSE

SsdA^{G103A}_v1 (Amino acid sequence):

KVSNIAESEAALGRASQARADLPQSKELKVKTVSSNDKKTLSGWGNKKPEGYERISAEQVKAKSEEIGH
EVKSHPYDRDYKGQYFSSHAEKQMSIASPNHPLAVSKPMCTDCQGYFSQLAKYSKVEQTVADPKAIRIF
KTDGSVETIMRSE

SsdA^{G103R}_v1 (Amino acid sequence):

KVSNIAESEAALGRASQARADLPQSKELKVKTVSSNDKKTLSGWGNKKPEGYERISAEQVKAKSEEIGH
EVKSHPYDRDYKGQYFSSHAEKQMSIASPNHPLRVSKPMCTDCQGYFSQLAKYSKVEQTVADPKAIRIF
KTDGSVETIMRSE

SsdA^{G103C}_v1 (Amino acid sequence):

KVSNIAESEAALGRASQARADLPQSKELKVKTVSSNDKKTLSGWGNKKPEGYERISAEQVKAKSEEIGH
EVKSHPYDRDYKGQYFSSHAEKQMSIASPNHPLCVSKPMCTDCQGYFSQLAKYSKVEQTVADPKAIRIF
KTDGSVETIMRSE

SsdA^{G103S}-T1 (Amino acid sequence, 127 aa):

SKELKVKTVSSNDKKTLSGWGNKKPEGYERISAEQVKAKSEEIGHEVKSHPYDRDYKGQYFSSHAEKQM
SIASPNHPLSVSKPMCTDCQGYFSQLAKYSKVEQTVADPKAIRIFKTDGSVETIMRSE

SsdA^{G103S}-T2 (Amino acid sequence, 121 aa):

KTVSSNDKKTLSGWGNKKPEGYERISAEQVKAKSEEIGHEVKSHPYDRDYKGQYFSSHAEKQMSIASPN
HPLSVSKPMCTDCQGYFSQLAKYSKVEQTVADPKAIRIFKTDGSVETIMRSE

SsdA^{G103S}-T3 (Amino acid sequence, 114 aa):

KKTLSGWGNKKPEGYERISAEQVKAKSEEIGHEVKSHPYDRDYKGQYFSSHAEKQMSIASPNHPLSVSK
PMCTDCQGYFSQLAKYSKVEQTVADPKAIRIFKTDGSVETIMRSE

SsdA^{G103S}-T4 (Amino acid sequence, 139 aa):

KVSNIAESEAALGRASQARADLPQSKELKVKTVSSNDKKTLSGWGNKKPEGYERISAEQVKAKSEEIGH
EVKSHPYDRDYKGQYFSSHAEKQMSIASPNHPLSVSKPMCTDCQGYFSQLAKYSKVEQTVADPKAIRIF
K

SsdA^{G103S}-T5 (Amino acid sequence, 102 aa):

KKTLSGWGNKKPEGYERISAEQVKAKSEEIGHEVKSHPYDRDYKGQYFSSHAEKQMSIASPNHPLSVSK
PMCTDCQGYFSQLAKYSKVEQTVADPKAIRIFK

SsdA^{G103S}_v1-CBE:

ATGAAGAGGACCGCCGATGGCTCTGAGTTCGAGAGCCCCAAGAAGAAGCGGAAGGTAATGAAGGTGTCA
AATATTGCGGAGTCCGAAGCTGCGCTTGGTCGTGCATCACAGGCCAGAGCCGACCTTCCGCAAAGTAAA
GAGTTGAAGGTAAAAACTGTTTCATCTAATGATAAGAAAACATTAAGTGGTTGGGGTAATAAAAAGCCC
GAAGGTAAGTCTTACTCTCTCTTTTTTGGTCTGTATTTTTAATTTTTTGAAGTATACTATTTGTACTGA
CGCTAATAATCTTTTTTCGCAGGTTATGAGAGGATTTCTGCGGAGCAGGTCAAGGCTAAGTCTGAAGAA
ATTGGGCATGAGGTGAAATCACACCCATACGACAGAGATTACAAAGGGCAGTACTTTTCATCTCATGCA
GAGAAACAGATGTCAATCGCAAGCCCAAATCATCCTTTAAGTGTTTCGAAGCCTATGTGTACTGATTGT
CAGGGGTACTTTTCTCAGCTGGCTAAGTATAGTAAGGTTGAGCAGACAGTAGCAGATCCTAAAGCGATA
CGTATTTTTAAGACCGATGGTTCTGTCGAAACAATTATGAGGTCGGAATCTAATTCGTCCGGCGGATCT
AGCGGAGGATCTAGCGGCTCCGAGACACCAGGAACATCCGAATCCGCTACACCAGAGTCGTCTGGAGGA
TCTAGCGGAGGATCTGACAAGAAGTACTCCATTGGGCTCGCGATCGGCACAAACAGCGTCGGCTGGGCC
GTCATTACGGACGAGTACAAGGTGCCGAGCAAAAAATTCAAAGTTCTGGGCAATACCGATCGCCACAGC
ATAAAGAAGAACCTCATTGGCGCCCTCCTGTTCGACTCCGGGGAGACGGCCGAAGCCACGCGGCTCAAA
AGAACAGCACGGCGCAGATATACCCGCAGAAAGAATCGGATCTGCTACCTGCAGGAGATCTTTAGTAAT
GAGATGGCTAAGGTGGATGACTCTTTCTTCCATAGGCTGGAGGAGTCCTTTTTGGTGGAGGAGGATAAA
AAGCACGAGCGCCACCCAATCTTTGGCAATATCGTGGACGAGGTGGCGTACCATGAAAAGTACCCAACC
ATATATCATCTGAGGAAGAAGCTTGTAGACAGTACTGATAAGGCTGACTTGCGGTTGATCTATCTCGCG
CTGGCGCATATGATCAAATTTCGGGGACACTTCCTCATCGAGGGGGACCTGAACCCAGACAACAGCGAT
GTCGACAAACTCTTTATCCAACTGGTTCAGACTTACAATCAGCTTTTCGAAGAGAACCCGATCAACGCA
TCCGGAGTTGACGCCAAAGCAATCCTGAGCGCTAGGCTGTCCAAATCCCGGCGGCTCGAAAACCTCATC
GCACAGCTCCCTGGGGAGAAGAAGAACGGCCTGTTTGGTAATCTTATCGCCCTGTCACTCGGGCTGACC
CCCAACTTTAAATCTAACTTCGACCTGGCCGAAGATGCCAAGCTTCAACTGAGCAAAGACACCTACGAT
GATGATCTCGACAATCTGCTGGCCCAGATCGGCGACCAGTACGCAGACCTTTTTTTGGCGGCAAAGAAC
CTGTCAGACGCCATTCTGCTGAGTGATATTCTGCGAGTGAACACGGAGATCACCAAAGCTCCGCTGAGC
GCTAGTATGATCAAGCGCTATGATGAGCACCACCAAGACTTGACTTTGCTGAAGGCCCTTGTCAGACAG
CAACTGCCTGAGAAGTACAAGGAAATTTTCTTCGATCAGTCTAAAAATGGCTACGCCGGATACATTGAC
GGCGGAGCAAGCCAGGAGGAATTTTACAAATTTATTAAGCCCATCTTGGAAAAAATGGACGGCACCGAG
GAGCTGCTGGTAAAGCTTAACAGAGAAGATCTGTTGCGCAAACAGCGCACTTTCGACAATGGAAGCATC
CCCCACCAGATTCACCTGGGCGAACTGCACGCTATCCTCAGGCGGCAAGAGGATTTCTACCCCTTTTTG
AAAGATAACAGGGAAAAGATTGAGAAAATCCTCACATTTCGGATACCCTACTATGTAGGCCCCCTCGCC
CGGGGAAATTCCAGATTCGCGTGGATGACTCGCAAATCAGAAGAGACTATCACTCCCTGGAACTTCGAG
GAAGTCGTGGATAAGGGGGCCTCTGCCCAGTCCTTCATCGAAAGGATGACTAACTTTGATAAAAATCTG
CCTAACGAAAAGGTGCTTCCTAAACACTCTCTGCTGTACGAGTACTTCACAGTTTATAACGAGCTCACC
AAGGTCAAATACGTCACAGAAGGGATGAGAAAGCCAGCATTCCTGTCTGGAGAGCAGAAGAAAGCTATC
GTGGACCTCCTCTTCAAGACGAACCGGAAAGTTACCGTGAAACAGCTCAAAGAAGATTATTTCAAAAAG
ATTGAATGTTTCGACTCTGTTGAAATCAGCGGAGTGGAGGATCGCTTCAACGCATCCCTGGGAACGTAT
CACGATCTCCTGAAAATCATTAAAGACAAGGACTTCCTGGACAATGAGGAGAACGAGGACATTCTTGAG
GACATTGTCCTCACCCTTACGTTGTTTGAAGATAGGGAGATGATTGAAGAACGCTTGAAAACTTACGCT
CATCTCTTCGACGACAAAGTCATGAAACAGCTCAAGAGGCGCCGATATACAGGATGGGGGCGGCTGTCA
AGAAAACTGATCAATGGGATCCGAGACAAGCAGAGTGGAAAGACAATCCTGGATTTTCTTAAGTCCGAT

GGATTTGCCAACCGGAACTTCATGCAGTTGATCCATGATGACTCTCTCACCTTTAAGGAGGACATCCAG
AAAGCACAAGTTTCTGGCCAGGGGGACAGTCTCCACGAGCACATCGCTAATCTTGCAGGTAGCCCAGCT
ATCAAAAAGGGAATACTGCAGACCGTTAAGGTCGTGGATGAACTCGTCAAAGTAATGGGAAGGCATAAG
CCCGAGAATATCGTTATCGAGATGGCCCGAGAGAACCAAACTACCCAGAAGGGACAGAAGAACAGTAGG
GAAAGGATGAAGAGGATTGAAGAGGGTATAAAAGAACTGGGGTCCCAAATCCTTAAGGAACACCCAGTT
GAAAACACCCAGCTTCAGAATGAGAAGCTCTACCTGTACTACCTGCAGAACGGCAGGGACATGTACGTG
GATCAGGAACTGGACATCAATCGGCTCTCCGACTACGACGTGGATCATATCGTGCCCCAGTCTTTTCTC
AAAGATGATTCTATTGATAATAAAGTGTTGACAAGATCCGATAAAAATAGAGGGAAGAGTGATAACGTC
CCCTCAGAAGAAGTTGTCAAGAAAATGAAAAATTATTGGCGGCAGCTGCTGAACGCCAAACTGATCACA
CAACGGAAGTTCGATAATCTGACTAAGGCTGAACGAGGTGGCCTGTCTGAGTTGGATAAAGCCGGCTTC
ATCAAAAGGCAGCTTGTTGAGACACGCCAGATCACCAAGCACGTGGCCCAAATTCTCGATTCACGCATG
AACACCAAGTACGATGAAAATGACAAACTGATTCGAGAGGTGAAAGTTATTACTCTGAAGTCTAAGCTG
GTTTCAGATTTCAGAAAGGACTTTCAGTTTTATAAGGTGAGAGAGATCAACAATTACCACCATGCGCAT
GATGCCTACCTGAATGCAGTGGTAGGCACTGCACTTATCAAAAAATATCCCAAGCTTGAATCTGAATTT
GTTTACGGAGACTATAAAGTGTACGATGTTAGGAAAATGATCGCAAAGTCTGAGCAGGAAATAGGCAAG
GCCACCGCTAAGTACTTCTTTTACAGCAATATTATGAATTTTTTCAAGACCGAGATTACACTGGCCAAT
GGAGAGATTCGGAAGCGACCACTTATCGAAACAAACGGAGAAACAGGAGAAATCGTGTGGGACAAGGGT
AGGGATTTCGCGACAGTCCGGAAGGTCCTGTCCATGCCGCAGGTGAACATCGTTAAAAAGACCGAAGTA
CAGACCGGAGGCTTCTCCAAGGAAAGTATCCTCCCGAAAAGGAACAGCGACAAGCTGATCGCACGCAAA
AAAGATTGGGACCCCAAGAAATACGGCGGATTCGATTCTCCTACAGTCGCTTACAGTGTACTGGTTGTG
GCCAAAGTGGAGAAAGGGAAGTCTAAAAAACTCAAAAGCGTCAAGGAACTGCTGGGCATCACAATCATG
GAGCGATCAAGCTTCGAAAAAAACCCCATCGACTTTCTCGAGGCGAAAGGATATAAAGAGGTCAAAAAA
GACCTCATCATTAAGCTTCCCAAGTACTCTCTCTTTGAGCTTGAAAACGGCCGGAAACGAATGCTCGCT
AGTGCGGGCGAGCTGCAGAAAGGTAACGAGCTGGCACTGCCCTCTAAATACGTTAATTTCTTGTATCTG
GCCAGCCACTATGAAAAGCTCAAAGGATCTCCCGAAGATAATGAGCAGAAGCAGCTGTTCGTGGAACAA
CACAAACACTACCTTGATGAGATCATCGAGCAAATAAGCGAATTCTCCAAAAGAGTGATCCTCGCCGAC
GCTAACCTCGATAAGGTGCTTTCTGCTTACAATAAGCACAGGGATAAGCCCATCAGGGAGCAGGCAGAA
AACATTATCCACTTGTTTACTCTGACCAACTTGGGCGCGCCTGCAGCCTTCAAGTACTTCGACACCACC
ATAGACAGAAAGCGGTACACCTCTACAAAGGAGGTCCTGGACGCCACACTGATTCATCAGTCAATTACG
GGGCTCTATGAAACAAGAATCGACCTCTCTCAGCTCGGTGGAGACAGCAGGGCTGACCCCAAGAAGAAG
AGGAAGGTGTTCGGCACCAACCTGTCCGACATCATCGAGAAGGAGACGGGCAAGCAACTCGTGATCCAG
GAGAGCATCCTCATGCTGCCAGAGGAGGTGGAGGAGGTCATCGGCAACAAGCCAGAGTCCGACATCCTG
GTGCACACCGCCTACGACGAGTCCACCGACGAGAACGTCATGCTCCTGACCAGCGACGCCCCAGAGTAC
AAGCCATGGGCCCTCGTCATCCAGGACAGCAACGGGGAGAACAAGATCAAGATGCTGTCGGGGGGGAGC
*CCAAAGAAGAAGCGGAAGGTG*TAG

HucoSsdA^{G103}-v1-CBE (human codon optimized):

bpNLS-SsdA^{G103}-linker-nCas9-NLS-UGI-NLS-T2A-dTomato

atgaagagaacagccgatggcagcgagttcgagagccccaagaaaaagcggaaagtgATGAAGGTGTCC
AATATCGCCGAGAGCGAGGCCGCTCTTGGCAGAGCTTCTCAGGCTAGAGCTGACCTGCCTCAGAGCAAA
GAGCTGAAAGTCAAGACCGTGTCCAGCAACGACAAGAAAACCCTGAGCGGCTGGGGCAACAAGAAGCCC
GAGGGCTACGAGAGAATCAGCGCCGAGCAAGTGAAGGCCAAGTCCGAAGAGATCGGCCACGAAGTGAAG
TCTCACCCCTACGACAGAGACTACAAGGGCCAGTACTTCAGCAGCCACGCCGAGAAGCAGATGTCTATC
GCCTCTCCTAATCACCCTCTGAGCGTGTCCAAGCCTATGTGCACCGATTGCCAGGGCTACTTCTCCCAG
CTGGCCAAGTACAGCAAGGTGGAACAGACCGTGGCTGACCCCAAGGCCATCAGAATCTTCAAGACCGAC
GGCAGCGTGGAAACCATCATGCGGAGCGAGagcaatagcagcggcggatctagcggaggcagctctgga
tctgagacacctggcacaagcgagagcgccacacctgaaagttctggcggttcttctggcggcagcgac
aagaagtactctatcggactggccatcggcaccaactctgttggatgggccgtgatcaccgacgagtac
aaggtgcccagcaagaaattcaaggtgctgggcaacaccgaccggcacagcatcaagaagaatctgatc
ggcgccctgctgttcgactctggcgaaacagccgaagccaccagactgaagaggactgcccgcagacgg
tacaccagaagaaagaaccggatctgctacctgcaagagatcttcagcaacgagatggccaaggtggac
gacagcttcttccacagactggaagagtccttcctggtggaagaggataagaagcacgagcggcacccc
atcttcggcaacatcgtggatgaggtggcctaccacgagaagtaccccaccatctaccacctgagaaag
aaactggtggacagcaccgacaaggccgacctgagactgatctatctggccctggctcacatgatcaag
ttccggggccacttcctgatcgagggcgacctgaatcctgacaacagcgacgtggacaagctgttcatc
cagctggtgcagacctacaaccagctgttcgaggaaaaccccatcaacgccagcggagtggacgccaaa
gccatcctgtctgccagactgagcaagagcagacggctggaaaacctgatcgctcagctgcccggcgag
aagaagaatggcctgttcggcaacctgattgccctgagcctgggcctgacacctaacttcaagagcaac
ttcgacctggccgaggacgccaagctccagctgtctaaggacacctacgacgacgacctggacaacctg
ctggcccagatcggagatcagtacgccgatctgtttctggccgccaagaacctgtccgacgccatcctg
ctgagcgacatcctgagagtgaacaccgagatcacaaaggccccactgagcgcctctatgatcaagaga
tacgacgagcaccaccaggatctgaccctgctgaaggccctcgttagacagcagctgccagagaagtac
aaagagattttcttcgaccagagcaagaacggctacgccggctacattgatggcggagccagccaagaa
gagttctacaagttcatcaagcccatcctggaaaagatggacggcaccgaggaactgctggtcaagctg
aacagagaggacctgctgcggaagcagcggaccttcgacaatggctctatccctcaccagatccacctg
ggagagctgcacgccattctgcggagacaagaggacttctacccattcctgaaggacaaccgggaaaag
atcgagaagatcctgaccttcagaatcccctactacgtgggccctctggccaggggaaatagcagattt
gcctggatgacccggaagtccgaggaaacaatcaccccttggaacttcgaggaagtggtggacaagggc
gccagcgctcagagcttcatcgagcggatgaccaacttcgataagaacctgcctaacgagaaggtgctg
cccaagcacagcctgctgtacgagtacttcaccgtgtataacgagctgaccaaagtgaaatacgtgacc
gagggaatgagaaagcccgcctttctgagcggcgagcagaaaaaggccattgtggatctgctgttcaag
accaaccgcaaagtgaccgtgaagcagctgaaagaggactacttcaagaaaatcgagtgcttcgacagc
gtcgagatcagcggcgtggaagatcggttcaatgccagcctgggcacataccacgacctgctgaaaatt
atcaaggacaaggacttcctggacaatgaggaaaacgaggacatccttgaggacatcgtgctgaccctg
acactgtttgaggacagagagatgatcgaggaacggctgaaaacatacgcccacctgttcgacgacaaa
gtcatgaagcaactgaagcggcggagatacaccggatggggcagactgtcccggaagctgatcaacggc

atccgggataagcagtccggcaagacaatcctggatttcctgaagtccgacggcttcgccaaccggaac
ttcatgcagctgatccacgacgacagcctgacctttaaagaggatatccagaaagcccaggtgtccggc
cagggcgattctctgcatgagcacattgccaacctggccggctctcccgccattaagaaaggcatcctg
cagacagtgaaggtggtggacgagctggtcaaagttatgggcagacacaagcccgagaacatcgtgatc
gaaatggccagagagaaccagaccacacagaagggacagaagaactcccgcgagagaatgaagcggatc
gaagagggcatcaaagaactgggcagccagatcctgaaagaacaccccgtggaaaacacccagctgcag
aacgagaagctgtacctgtactacctgcagaatggacgggatatgtacgtggaccaagagctggacatc
aaccggctgtccgactacgatgtggaccatatcgtgccccagagctttctgaaggacgactccatcgac
aacaaagtgctgaccagatccgacaagaatcggggcaagagcgacaacgtgccctccgaagaggtggtc
aagaagatgaagaactactggcgacagctgctgaatgccaagctgattacccagcggaagttcgataac
ctgaccaaggccgagagaggcggcctgtctgaactggataaggccggcttcatcaagaggcagctggtg
gaaacccggcagatcaccaaacacgtggcacagattctggactcccggatgaacactaagtacgacgag
aacgacaaactgatccgggaagtgaaagtgatcaccctcaagtccaagctggtgtccgactttcggaag
gatttccagttttacaaagtgcgcgagatcaacaactaccatcacgcccacgacgcctacctgaatgcc
gttgttggaacagccctgatcaagaagtatcccaagctggaatccgagttcgtgtacggcgattacaag
gtgtacgacgtgcggaagatgatcgccaaaagcgagcaagagattggcaaggctaccgccaagtacttt
ttctacagcaacatcatgaactttttcaagacagagatcacgctggccaacggcgagatcagaaagcgg
cctctgatcgagacaaacggcgaaaccggcgagattgtgtgggataagggcagagactttgccacagtg
cgaaaggtgctgagcatgccccaagtgaatatcgtgaagaaaaccgaggtgcagacaggcggcttcagc
aaagagtctatcctgcctaagcggaactccgacaagctgatcgcccggaagaaggactgggaccctaag
aagtacggcggcttcgatagccctaccgtggcctattctgtgctggtggtggccaaagtggaaaagggc
aagtccaaaaagctcaagagcgtgaaagagctgctggggatcaccatcatggaaagaagcagcttcgag
aagaatcccatcgactttcttgaggccaagggctacaaagaagtcaagaaggacctgatcatcaagctc
cccaagtactccctgttcgagctggaaaatggccggaagcggatgctggcttctgctggcgaactgcag
aagggcaacgaactggccctgcctagcaaatatgtgaacttcctgtacctggcctctcactatgagaag
ctgaagggcagccccgaggataatgagcaaaagcagctgtttgtggaacagcacaagcactacctggac
gagatcatcgagcagatctccgagttctccaagagagtgatcctggccgacgctaatctggacaaggtc
ctgtccgcctacaacaagcaccgggacaagcctatcagagagcaggccgagaatatcatccacctgttt
accctgaccaacctgggagcccctgccgccttcaagtacttcgacaccaccatcgaccggaagcgctac
accagcaccaaagaggtgctggacgccacactgatccaccagtctatcaccggcctgtacgagacacgg
atcgacctgtctcagctcggcggcat
tctagagccgatcctaaaaagaaacggaaggtgttcggcacaaacctcagcgacatcatcgagaaagag
acaggcaagcagctggtcatccaagaatccatcctgatgctgcctgaagaggttgaggaagtgatcggc
aacaagcctgagagcgatatcctggtgcacaccgcctacgatgagagcaccgatgagaacgtgatgctg
ctgacaagcgacgcccctgagtacaagccttgggctctcgtgatccaggacagcaatggcgagaacaag
atcaagatgctgtccggcggcagccccaaaaagaagagaaaagtcactagtggcGAGGGCAGAGGCTCC
CTGCTGACATGCGGAGATGTGGAAGAGAATCCTGGCCCCagc**ATGGTGTCCAAGGGGGAGGAAGTGATC**
AAAGAATTCATGCGGTTCAAAGTGCGGATGGAAGGCAGCATGAACGGCCACGAGTTCGAGATCGAAGGC
GAGGGCGAAGGCAGACCCTACGAGGGAACACAGACCGCCAAACTGAAAGTGACAAAGGGCGGACCCCTG
CCCTTCGCCTGGGATATCCTGAGCCCCCAGTTTATGTACGGCAGCAAGGCCTACGTGAAGCACCCCGCC
GACATCCCCGATTACAAGAAGCTGAGCTTCCCCGAGGGCTTCAAGTGGGAAAGAGTGATGAATTTCGAG
GACGGCGGCCTCGTGACAGTGACCCAGGATAGCTCTCTGCAAGACGGCACACTGATCTACAAAGTGAAG

ATGCGGGGCACCAATTTCCCCCCCGACGGCCCCGTGATGCAGAAAAAAACCATGGGATGGGAGGCCTCT
ACCGAGCGGCTGTACCCTAGAGATGGCGTGCTGAAGGGGGAAATCCATCAGGCCCTGAAGCTGAAAGAC
GGCGGCCACTACCTGGTGGAATTCAAGACCATCTACATGGCCAAGAAACCCGTGCAGCTGCCAGGCTAC
TATTACGTGGACACAAAGCTGGATATCACCAGCCACAACGAGGACTACACAATCGTGGAACAGTACGAG
CGGAGCGAGGGGCGGCACCATCTGTTTCTGTATGGCATGGATGAGCTGTACAAATGA
